# Advances in photoacoustic imaging reconstruction and quantitative analysis for biomedical applications

**DOI:** 10.1186/s42492-025-00213-x

**Published:** 2026-02-01

**Authors:** Lei Wang, Weiming Zeng, Kai Long, Hongyu Chen, Rongfeng Lan, Li Liu, Wai Ting Siok, Nizhuan Wang

**Affiliations:** 1 The Laboratory of Digital Image and Intelligent Computation, Shanghai Maritime University, Shanghai 201306, China; 2 School of Electronic Engineering and Intelligent Manufacturing, Anqing Normal University, Anqing 246133, Anhui China; 3 School of Engineering, Great Bay University, Dongguan 523000, Guangdong China; 4 Department of Cell Biology & Medical Genetics, School of Basic Medical Sciences, Shenzhen University Medical School, Shenzhen 518060, Guangdong China; 5 Department of Chinese and Bilingual Studies, The Hong Kong Polytechnic University, Hong Kong 999077, China

**Keywords:** Photoacoustic imaging, Deep learning, Photoacoustic image reconstruction, Quantitative analysis

## Abstract

Photoacoustic imaging (PAI), a modality that combines the high contrast of optical imaging with the deep penetration of ultrasound, is rapidly transitioning from preclinical research to clinical practice. However, its widespread clinical adoption faces challenges such as the inherent trade-off between penetration depth and spatial resolution, along with the demand for faster imaging speeds. This review comprehensively examines the fundamental principles of PAI, focusing on three primary implementations: photoacoustic computed tomography, photoacoustic microscopy, and photoacoustic endoscopy. It critically analyzes their respective advantages and limitations to provide insights into practical applications. The discussion then extends to recent advancements in image reconstruction and artifact suppression, where both conventional and deep learning (DL)-based approaches have been highlighted for their role in enhancing image quality and streamlining workflows. Furthermore, this work explores progress in quantitative PAI, particularly its ability to precisely measure hemoglobin concentration, oxygen saturation, and other physiological biomarkers. Finally, this review outlines emerging trends and future directions, underscoring the transformative potential of DL in shaping the clinical evolution of PAI.

## Introduction

### Fundamentals of photoacoustic imaging

Optical imaging and ultrasound imaging (US) are well-established modalities that play complementary roles in medical diagnostics [1]. Optical imaging offers high sensitivity and intrinsic molecular contrast but is fundamentally limited by strong optical scattering in biological tissues, which restricts high-resolution imaging to superficial depths [2]. In contrast, US provides excellent spatial resolution at depths of several centimeters owing to low acoustic scattering, making it indispensable in cardiovascular, abdominal, and obstetric applications [3, 4]. However, US suffers from low intrinsic contrast because soft tissues exhibit minimal differences in acoustic impedance, and conventional US contrast agents (e.g., microbubbles) do not enhance optical absorption. This limitation has motivated the development of hybrid imaging approaches that combine the rich optical contrast of absorption-based imaging with deep penetration and high-resolution US [5–7].

**Fig. 1:**
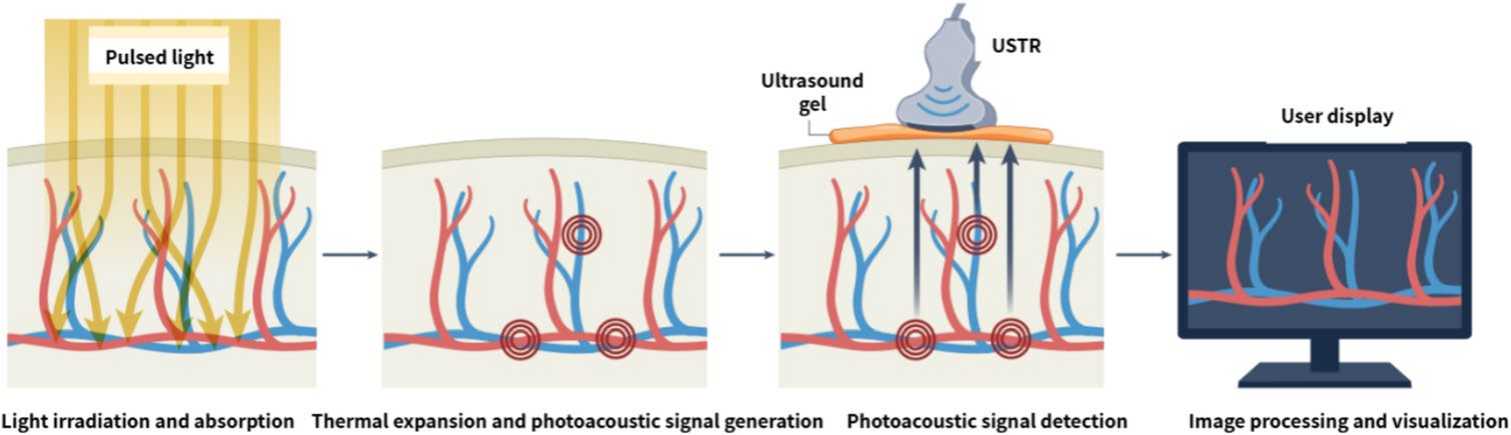
Schematic of photoacoustic image generation process [7]

Photoacoustic imaging (PAI) utilizes the photoacoustic effect, a hybrid physical process in which pulsed laser light is absorbed by tissue chromophores, converted into a transient temperature increase, and subsequently generates ultrasonic waves through thermoelastic expansion [7, 8]. As illustrated in Fig. 1, optical excitation leads to selective absorption by endogenous chromophores–such as oxy- and deoxy-hemoglobin, melanin, lipids, collagen, DNA, and RNA–or by exogenous agents, including clinically approved dyes such as indocyanine green (ICG) and methylene blue. The resulting localized thermal expansion induces broadband acoustic waves that propagate to the tissue surface, where they are detected using an US transducer array. By analyzing the time-of-flight and amplitude of these signals, a two- or three-dimensional (3D) map of the initial optical absorption distribution can be reconstructed, providing high-resolution visualization of both functional and molecular tissue characteristics [7, 9].

While small-molecule dyes, such as ICG, offer clinical compatibility, their limited photostability, narrow absorption tunability, and rapid renal clearance restrict their utility in advanced molecular imaging. Engineered nanomaterial-based photoacoustic agents have emerged as promising alternatives to address these limitations. Among them, gold nanostructures (e.g., nanorods and nanocages) leverage localized surface plasmon resonance to achieve strong and spectrally tunable near-infrared absorption, enabling high-contrast imaging and photothermal therapy. However, their clinical translation is hindered by potential long-term accumulation in the reticuloendothelial system (e.g., liver and spleen) and slow biodegradation, raising concerns about chronic toxicity. In contrast, semiconducting polymer nanoparticles exhibit excellent photostability, high molar extinction coefficients in the NIR-II window, and favorable biodegradability profiles, with several formulations demonstrating efficient renal and hepatobiliary clearance. Perovskite nanocrystals offer ultrahigh absorption coefficients and sharp spectral features; however, they face significant biosafety barriers owing to their lead content, poor colloidal stability in physiological environments, and risk of releasing toxic ions upon degradation. Collectively, the clinical adoption of these nanoplatforms requires rigorous assessment of biocompatibility, clearance kinetics, and regulatory compliance–particularly for agents containing heavy metals or non-biodegradable components [10–12].

The efficient generation of photoacoustic signals, irrespective of the absorber type, relies on two key physical conditions: stress confinement and thermal confinement. Stress confinement requires the laser pulse duration to be shorter than the stress relaxation time (i.e., the time required for the pressure to propagate out of the irradiated volume), ensuring that the induced pressure is not dissipated during laser excitation. Thermal confinement requires negligible heat diffusion during the pulse so that the energy remains localized. Under these conditions, the fractional volume change is governed by the following thermoelastic equation:

1 $$\begin{aligned} \frac{\Delta V}{V} = -\kappa \Delta p + \beta \Delta T \end{aligned}$$

where $\Delta p$ and $\Delta T$ are the changes in pressure and temperature, respectively; $\kappa$ is the isothermal compressibility; and $\beta$ is the volumetric thermal expansion coefficient [13]. The initial pressure increase $p_0$ is directly proportional to the absorbed optical energy density $E_a = \mu _a F$, yielding

2 $$\begin{aligned} p_0 = \frac{\beta }{\kappa \rho c_v} \eta _{\text {th}} \mu _a F = \Gamma \eta _{\text {th}} \mu _a F = \Gamma E_a \end{aligned}$$

where $\rho$ is the mass density, $c_v$ is the specific heat capacity at constant volume, $\eta _{\text {th}}$ is the thermal conversion efficiency (typically close to unity for nonradiative absorbers), $\mu _a$ is the absorption coefficient, *F* is the optical fluence, and $\Gamma = \beta / (\kappa \rho c_v)$ is the dimensionless Grüneisen parameter. This linear relationship forms the basis for quantitative PAI, enabling the estimation of chromophore concentration and oxygen saturation.

### Advances of PAI

PAI overcomes the depth limitations of conventional optical imaging by converting absorbed light into detectable US, thereby enabling high-resolution imaging at depths of approximately 1 cm or greater in scattering tissues [14]. This hybrid modality facilitates the label-free in vivo visualization of morphological, physiological, and metabolic processes [15]. For instance, the distinct absorption spectra of oxyhemoglobin and deoxyhemoglobin allow PAI to non-invasively quantify blood oxygen saturation ($\textrm{sO}_2$), a key functional biomarker in oncology and neuroscience [16].

**Fig. 2:**
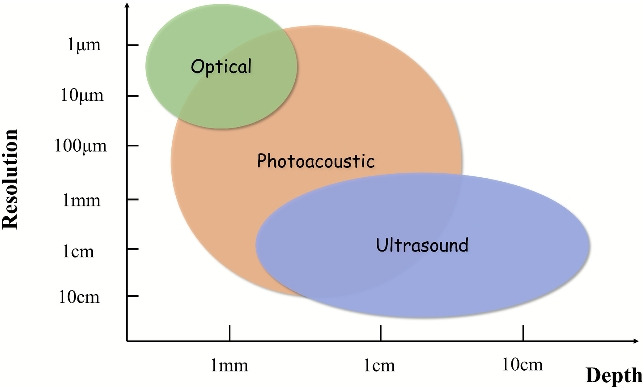
Bridging the imaging gap: comparison of resolution versus depth in photoacoustic, ultrasound, and optical imaging

The characteristic performance envelopes of optical coherence tomography (OCT), PAI, and US in terms of their fundamental resolution-depth trade-offs are conceptually illustrated in Fig. 2. The approximate ranges shown are representative and well-established in the literature: Optical imaging (e.g., OCT) provides high resolution but is limited to superficial depths; US achieves deep penetration at the expense of resolution [17], whereas PAI uniquely bridges these two regimes, enabling high-sensitivity optical contrast at ultrasonic scales [18–20]. As summarized in Table 1, PAI uniquely bridges the gap between optical and US; it provides optical-absorption-based contrast superior to US, with a penetration depth far exceeding that of OCT. A key advantage is that PAI does not rely on coherent light detection, thus avoiding inherent coherent speckle noise that degrades OCT images. It is noteworthy that while higher US frequencies improve the spatial resolution, they concomitantly reduce the imaging depth owing to increased acoustic attenuation [21].

Despite challenges such as optical fluence heterogeneity and reconstruction complexity, PAI stands out for its non-invasiveness, absence of ionizing radiation, and molecular specificity, attributes that have driven rapid advances in hardware miniaturization, multispectral unmixing, and deep learning (DL)-based image reconstruction since PAI’s first in vivo demonstrations [20, 22], with recent advances further exemplified by ref. [23].

In particular, PAI has emerged as a powerful platform for functional and molecular neuroimaging. Its sensitivity to hemoglobin oxygenation enables noninvasive, label-free mapping of cerebral hemodynamics and $\textrm{sO}_2$ dynamics during neural activation, making it ideally suited for studying neurovascular coupling, the fundamental link between neuronal activity and the local hemodynamic response. Recent studies have integrated PAI with optogenetic or ultrasonic brain stimulation to probe causal relationships in neural circuits. Advances in high-speed imaging and motion-robust reconstruction now permit functional monitoring in awake, behaving animal models. These developments underscore PAI’s growing role in bridging molecular specificity with system-level neuroscience [24–26].

A prominent example of clinical translation is intravascular photoacoustic imaging (IVPAI). By integrating a miniaturized photoacoustic catheter with intravascular US, IVPAI enables co-registered structural and compositional imaging of arterial walls. Lipids exhibit strong absorption at characteristic wavelengths (e.g., 1210 and 1720 nm), allowing IVPAI to specifically identify lipid-rich necrotic cores–a hallmark of vulnerable atherosclerotic plaques. Recent clinical studies demonstrated the feasibility of IVPAI in human coronary arteries, whereas hybrid systems combined with multispectral unmixing and DL are paving the way for real-time automated plaque classification [27, 28].

Collectively, by uniquely integrating optical-absorption-based contrast with US-level penetration depth, PAI is poised to drive transformative advances across a broad spectrum of biomedical applications, from fundamental neuroscience and cancer research to clinical cardiology and real-time intraoperative guidance.

**Table 1:** Comparative analysis of OCT, US, and PAI

Characteristic	OCT	US	PAI (PAM/PACT)
Imaging depth (mm)	1–2	20–100	0.1–1 (PAM)/10–80 (PACT)
Axial resolution ($\upmu$m)	5–15	100–500	1–15 (PAM)/50–200 (PACT)
Lateral resolution ($\upmu$m)	5–20	200–1000	0.5–5 (PAM)/200–1000 (PACT)
Contrast mechanism	Optical scattering	Acoustic impedance	Optical absorption (hemoglobin, melanin, lipids, contrast agents)
Signal texture	Coherent speckle	Acoustic speckle	Minimal in well-defined absorbers such as blood vessels; texture-like patterns may appear in heterogeneous or scattering tissues
Functional contrast	Limited (angiography)	Moderate (Doppler, elastography)	High (sO_2_, total Hb, flow, metabolism)
Clinical adoption	Established (ophthalmology)	Widespread	Emerging (oncology, neurology, cardiovascular, dermatology)
Representative applications	Retinal imaging, intravascular coronary imaging	Abdominal organ imaging, fetal monitoring	Oncology (breast cancer, melanoma), neurology (functional brain imaging), cardiovascular (atherosclerotic plaque detection), dermatology (microvasculature mapping)

For PAI, values are given as PAM/PACT and represent typical performance. Values are approximate and depend on system configuration (e.g., transducer frequency, laser parameters)

*PAI* Photoacoustic imaging, *US* Ultrasound, *OCT* Optical coherence tomography, *PACT* Photoacoustic compute tomography, *PAM* Photoacoustic microscopy

## Key implementations of PAI

**Fig. 3:**
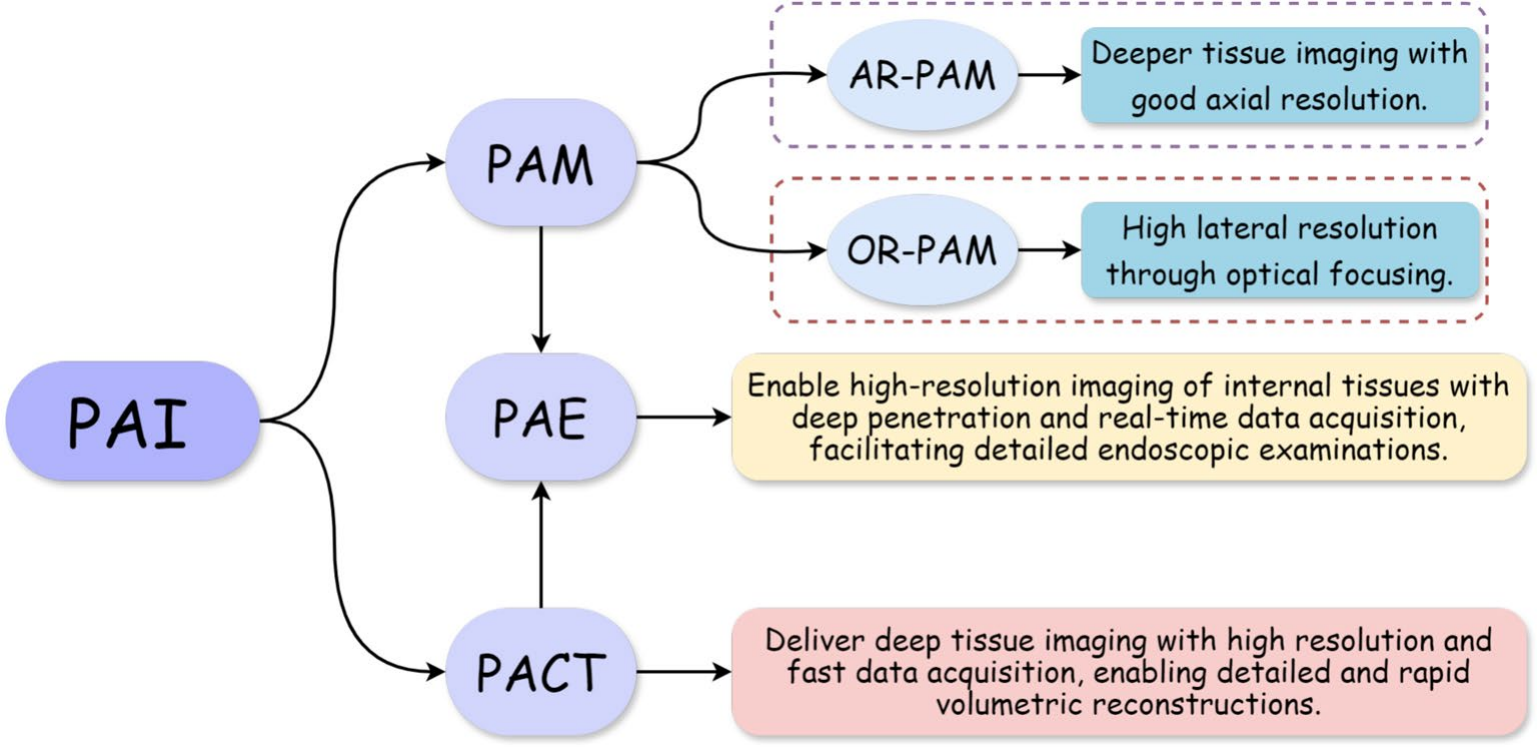
Three key implementations of PAI. PAI: Photoacoustic imaging; PACT: Photoacoustic compute tomography; PAM: Photoacoustic microscopy; PAE: Photoacoustic endoscopy; OR-PAM: Optical-resolution photoacoustic microscopy; AR-PAM: Acoustic-resolution photoacoustic microscopy

The study of the photoacoustic effect, spurred by advancements in laser technology from the late 1970 s to the early 1980 s, laid the foundation for PAI [16]. This groundwork culminated in the early 21st century with the maturation development of key implementations, such as photoacoustic compute tomography (PACT), photoacoustic microscopy (PAM), and photoacoustic endoscopy (PAE), which significantly advanced the field and expanded its biomedical applications, opening new avenues for research and clinical practice [29–31]. Figure 3 illustrates the three main methodologies employed in PAI.

### PACT

PACT is a rapidly advancing imaging modality that leverages the photoacoustic effect to provide high-contrast, non-ionizing imaging of biological tissues [32, 33]. It offers a wide field of view and deep penetration, enabling diverse biomedical applications such as whole-organ imaging, vascular mapping, and quantitative monitoring of blood oxygen saturation ($\textrm{sO}_2$) in cancer and neuroscience [15, 34].

In PACT, a wide-diameter unfocused pulsed laser beam illuminates the tissue surface, and the resulting photoacoustic signals are captured by an array of ultrasonic detectors [29]. Inversion algorithms process these signals to reconstruct the initial acoustic field, yielding high-resolution images at scales from the cellular to the organ level, with details down to hundreds of micrometers [35, 36]. PACT offers moderate spatial resolution, significant penetration depth, and fast imaging speed. The selection of an ultrasonic transducer array depends on the specific application. Linear arrays are suitable for small animals or surface tissues, whereas hemispherical arrays are better suited for broader human imaging, particularly for breast cancer detection [37, 38]. The potential use of PACT in breast cancer imaging has driven significant advancements in clinical research, leading to the development of multiple imaging systems [39]. Despite its advantages in deep-tissue imaging, the spatial resolution of PACT in biological tissues typically ranges from 50 to 150 $\upmu m$. This resolution is lower than that of OCT but comparable to or slightly finer than that of clinical US [40]. In addition, the contrast mechanisms differ between the macroscopic and microscopic imaging scales [22]. Table 2 summarizes PACT’s advantages and constraints in comparison to US, OCT, and conventional optical imaging modalities.

**Table 2:** Advantages and constraints of PACT modality in comparison with US, OCT, and conventional optical imaging techniques

Modality	Advantage	Constraint
PACT	Non-invasive and free from ionizing radiation	Higher system cost compared to US
	Wide field of view	Lower spatial resolution than OCT and high-frequency US
	Greater imaging depth than OCT and conventional optical imaging, enabling millimeter-to-centimeter scale imaging	Lacks subcellular resolution, limiting integration with high-resolution optical microscopy
	Fast imaging speed (enabling real-time or functional imaging)	
	Spatial resolution between that of US and OCT (typically 50–200$\upmu$m)	
	Versatile across cellular-to-organ scales	

*PACT* Photoacoustic compute tomography, *OCT* Optical coherence tomography, *US* Ultrasound

Recent advancements in PACT are driven by innovations in imaging devices, contrast agents, and models [41]. PACT relies on reconstruction algorithms to transform the detected photoacoustic signals into acoustic source distributions, thereby generating high-resolution images [42]. These algorithms play crucial roles in determining imaging accuracy and quality [43]. However, several challenges remain, including limited angular coverage and constrained detection bandwidth, both of which can degrade image resolution and contrast [42, 44]. The reconstruction process inherently involves solving an ill-posed inverse acoustic problem using incomplete data. To further enhance PACT’s utility in biomedical imaging, it is essential to optimize existing technologies and develop novel methodologies.

These technological and algorithmic advancements have paved the way for DL-enhanced PACT. Recent studies have demonstrated the potential of longitudinal tumor monitoring and therapy response evaluation. By leveraging PAI’s sensitivity to oxygenation and vascular dynamics, DL-based reconstruction enables the visualization of temporal changes in tumor hypoxia, angiogenesis, and metabolic reprogramming during immunotherapy and photothermal therapy. Furthermore, data-driven quantitative frameworks improve temporal consistency and allow predictive modeling of treatment efficacy, thereby highlighting the translational promise of PAI for noninvasive cancer therapy assessment [45].

**Table 3:** Comparative analysis of OR-PAM and AR-PAM modalities

Characteristic	OR-PAM	AR-PAM
**Advantage**		
Lateral resolution	High ($< 5 \upmu$m)	Moderate ($> 50 \upmu$m)
Axial resolution	Determined by US transducer bandwidth	Determined by US transducer bandwidth
Depth penetration	Limited ($\sim$1–2 mm)	Deeper ($\sim$3–10 mm)
Contrast mechanism	Enables high-resolution visualization of microvasculature and cellular structures	Suitable for functional imaging beyond 1 mm depth
Imaging precision	Suitable for high-precision surface imaging	Suitable for deeper tissue imaging
**Constraint**		
Depth penetration	Restricted by light scattering effects	Hindered by low resolution and high background noise
Lateral resolution	Limited applicability for deep tissue imaging	Lower resolution due to acoustic focusing
Imaging speed	Speed constrained by mechanical scanning	Slower due to broader imaging coverage
Contrast noise ratio	Challenged by reduced contrast in deeper tissues	Higher background noise impacting clarity
Clinical application	Constrained by depth and speed limitations	Challenged by lower resolution and noise in deeper tissues

*OR-PAM* Optical-resolution photoacoustic microscopy, *AR-PAM* Acousticresolution photoacoustic microscopy, *US* Ultrasound

### PAM

Over the past decade, PAM has emerged as a crucial technique for microscopic imaging owing to its distinctive optical absorption contrast mechanism [33, 36]. As a versatile biomedical imaging modality, PAM facilitates structural, functional, and molecular imaging by using both endogenous and exogenous contrast agents. It has been widely applied in blood flow perfusion, oxygenation imaging, tumor visualization, and neuroimaging by leveraging hemoglobin as an intrinsic optical absorber [44, 46].

PAM is broadly categorized into optical-resolution photoacoustic microscopy (OR-PAM) and acoustic-resolution photoacoustic microscopy (AR-PAM) depending on whether the lateral resolution is governed by the optical or acoustic focus [5, 20, 47].

While PACT achieves centimeter-scale penetration depths, PAM–particularly OR-PAM–delivers a significantly higher spatial resolution (typically $<5 \upmu$m) in superficial tissues (1 mm to 2 mm), and a depth range accessible to both modalities. However, PAM has been optimized for surface and near-surface imaging [20, 39].

In OR-PAM, a tightly focused laser enables micron-scale lateral resolution but is limited to depths of 1 mm to 2 mm owing to optical scattering. In contrast, AR-PAM uses weakly focused or collimated illumination, which shifts the resolution limit to the acoustic focus (typically $> 50 \upmu$m) and enables greater imaging depths of 3 mm to 10 mm. However, a larger illuminated area can result in stronger background signals. In both configurations, the axial resolution is determined by the bandwidth of the US transducer. A key challenge in AR-PAM is improving the lateral resolution without sacrificing penetration depth.

Most OR-PAM systems rely on mechanical scanning for both optical excitation and US detection, which limits their imaging speeds. Nevertheless, OR-PAM remains invaluable for high-resolution biomedical applications, such as tumor angiography and melanoma cell imaging [37, 48]. AR-PAM, which overcomes the optical diffusion limit, is well suited for deeper functional imaging beyond 1 mm, enabling the quantification of oxygenated and deoxygenated hemoglobin concentrations, blood flow velocity, and oxygen metabolism rates [49].

One of the primary technical challenges in PAM is the optimization of the tradeoff between spatial resolution and imaging speed [48, 50]. Despite its potential, the clinical translation of PAM has been constrained by several interdependent challenges, including high laser excitation doses, limited imaging throughput, and difficulties in consistently achieving optimal image quality [22, 37]. Therefore, current research efforts are focused on developing advanced image and signal processing techniques to address these limitations [51]. Table 3 summarizes the advantages and constraints of PAM.

**Table 4:** Advantages and constraints of PAE modality

Characteristic	Advantage	Constraint
PAE	High optical contrast for superficial tissues	Limited lateral resolution (typically 50$\upmu$m 200$\upmu$m)
	Functional imaging capability (e.g., oxygen saturation, hemoglobin concentration)	Restricted field of view due to lumen geometry
	Compatibility with endoscopic platforms for *in vivo* use	Susceptibility to physiological motion artifacts (e.g., peristalsis, respiration)
	Potential for multimodal integration (e.g., with OCT or fluorescence)	Signal attenuation in highly scattering or absorbing luminal tissues
	Miniaturized probe design enables access to confined anatomical sites	Challenges in system miniaturization and signal-to-noise ratio optimization

*PAM* Photoacoustic microscopy, *OCT* Optical coherence tomography

### PAE

Conventional PACT and PAM are unsuitable for imaging internal structures such as the digestive tract and vascular walls. To overcome this limitation, researchers integrated photoacoustic sensing with endoscopic technology, giving rise to PAE for accessing deeper tissues [23, 52]. Although PAE has emerged more recently than PACT and PAM, it has rapidly advanced and can image internal tissues, such as the digestive tract, blood vessels, and the urogenital system, emerging as a significant area of research [52].

PAE is particularly suitable for detecting lesions in biological cavities, such as the nasal cavity, digestive tract, and arterial vessels, and offers high-quality images of superficial tissues for diagnostic purposes. It utilizes miniaturized MEMS scanning devices and fiber-optic sensors to reduce the system size, enabling in vivo imaging for the examination of organ conditions or lesions. However, spatial constraints within the lumen limit the angular range of the detector for collecting photoacoustic signals, which may compromise the image quality. Table 4 summarizes the advantages and constraints of PAE, and Table 5 compares the characteristics of the three principal PAI modalities.

Recently, PAE has shown remarkable potential for both preclinical and translational biomedical applications [14, 53, 54]. In gastrointestinal imaging, miniaturized photoacoustic endoscopes have been developed to map vascular morphology, oxygen saturation, and inflammatory responses in organs such as the esophagus, stomach, and colon [14, 53]. These systems enable the early detection of precancerous and neoplastic lesions by resolving microvascular networks with high optical contrast. In cardiovascular research, intravascular PAE systems have been utilized to characterize atherosclerotic plaques, lipid distribution, and vascular wall composition, offering morphological and molecular contrasts that complement traditional US and OCT. Moreover, prototype PAE probes have been applied to the urinary and reproductive tracts for the real-time visualization of mucosal structures and microvasculature, highlighting their potential for minimally invasive diagnosis and intraoperative monitoring of urogenital diseases [53, 55]. Collectively, these studies underscore the versatility of PAE as a cross-disciplinary imaging platform capable of providing structural and functional insights [14, 54].

Despite rapid progress, several challenges remain before PAE can achieve widespread clinical deployment [14, 53]. Miniaturization and system integration are constrained by the narrow diameter of biological lumens, which necessitates delicate trade-offs between spatial resolution, imaging depth, and field of view [53, 56]. In vivo imaging stability is further affected by physiological motions such as respiration and peristalsis, whereas limited-view detection geometries can lead to signal dropout and reconstruction artifacts. Overcoming these issues requires continued innovation in compact scanning mechanisms, flexible probe architectures, and computational reconstruction algorithms capable of compensating for incomplete acoustic sampling [54, 55, 57]. Emerging trends include the development of multimodal endoscopic systems that integrate PAE with OCT, fluorescence, or US, as well as DL-based frameworks for artifact correction and real-time reconstruction [54, 55, 58]. With continued advances in MEMS technology, fiber-optic detection, and intelligent reconstruction algorithms, PAE is expected to evolve into a clinically viable tool for early diagnosis, functional assessment, and intraoperative guidance in gastrointestinal, vascular, and urogenital applications [14, 53].

**Fig. 4:**
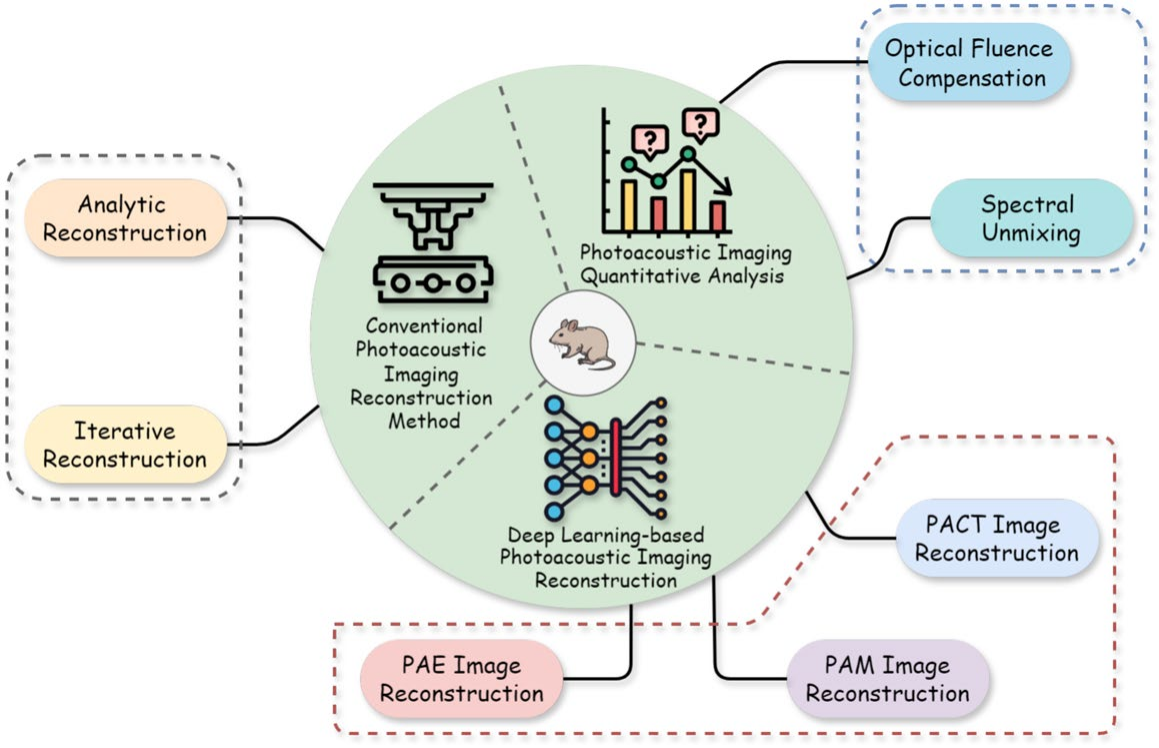
Illustrative overview outlining the scope of this review. PACT: Photoacoustic compute tomography; PAE: Photoacoustic endoscopy; PAM: Photoacoustic microscopy

The subsequent section delves into image reconstruction techniques for PAI, covering both conventional algorithms and DL-based frameworks, and provides a quantitative analysis of PAI, as outlined in Fig. 4.

**Table 5:** Comparison of characteristics among three PAI modalities

Modality	Resolution	Field of view	Depth	Cost
PACT	Low	Large	Deep	Expensive
AR-PAM	Moderate	Limited by scanning range	Moderate	Moderate
OR-PAM	High	Small	Shallow	Expensive
PAE	Moderate	Small	Shallow	Moderate

Resolution rankings are relative among PAI modalities. Shallow refers to depths$<2$ mm (e.g., skin, epidermis); Moderate refers to 2–10 mm (e.g., small animal organs); Deep refers to$>10$ mm up to several centimeters (e.g., breast, brain). Cost categories are qualitative: Expensive indicates systems requiring ultrafast lasers and high-end detection components (e.g., PACT, OR-PAM); Moderate refers to systems using standard or lower-cost components (e.g., AR-PAM, PAE)

*PACT* Photoacoustic compute tomography, *OR-PAM* Optical-resolution photoacoustic microscopy, *AR-PAM* Acoustic-resolution photoacoustic microscopy, *PAE* Photoacoustic endoscopy, *PAI* Photoacoustic imaging

**Fig. 5:**
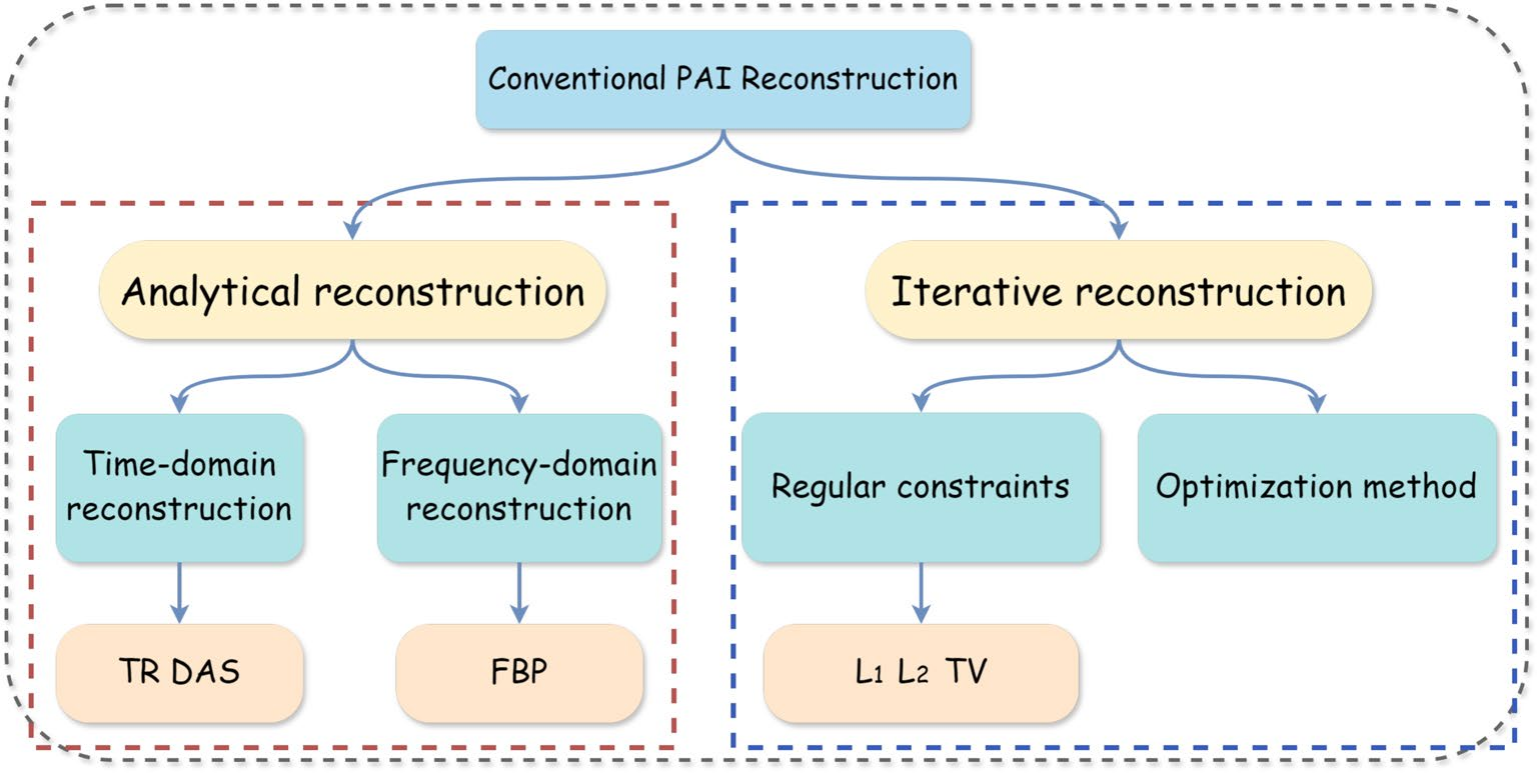
Illustration of conventional PAI reconstruction methods. PAI: Photoacoustic imaging; FBP: Filtered back projection; TR: Time reversal; DAS: Delay-and-sum

## Conventional reconstruction in PAI

The core principle of PAI is to map the optical absorption coefficient distribution within the tissues, which is directly correlated with the initial acoustic pressure distribution. Developing robust reconstruction models is crucial for accurately reconstructing the initial acoustic pressure from acquired photoacoustic signals [59].

Conventional reconstruction methods can be broadly classified into analytical and iterative methods [60] as shown in Fig. 5. Analytical reconstruction methods use time- and frequency-domain techniques to find optimal solutions, such as time reversal (TR) [61, 62], delay-and-sum (DAS) [63, 64] in the time domain, and filtered back projection (FBP) [65] in the frequency domain.

High-fidelity image reconstruction is vital for improving the accuracy of PAI because it converts raw signals from ultrasonic transducers into images of the initial pressure distributions [66]. When data acquisition (DAQ) is incomplete, conventional PAI reconstruction methods such as back projection [67], TR, and DAS may result in diminished image quality and depth [68].

Unlike analytical methods, iterative reconstruction uses optimization and regularization to solve equations, offering higher signal-to-noise ratios (SNRs) and improved image quality, but it requires more computational power. It assumes ideal conditions such as high sampling rates and uniform sound speed, which are often not met in real scenarios, leading to errors and degraded image quality. Although iterative methods can partially mitigate these issues, they require substantial computational resources and careful selection of regularization techniques [69].

Despite significant engineering efforts to address the technical challenges of PAI, most solutions rely on complex, expensive hardware and time-consuming image reconstruction processes, including iterative methods. These approaches often require balancing parameters, such as speed *vs* field of view and resolution *vs* penetration depth. Therefore, innovative approaches that address these challenges from a new perspective are required [70].

## DL-based reconstruction in PAI

DL has advanced rapidly, driven by large datasets and powerful computing resources, and is now crucial for tasks such as image classification, segmentation, reconstruction, superresolution, and disease prediction. PAI reconstruction surpasses traditional algorithms by generating high-quality images with enhanced SNR, even at low pulse energy levels [71]. Its integration into PAI significantly improves artifact removal, denoising, and super-resolution [38, 65, 68], and enhances image quality under non-ideal conditions, addressing challenges such as sparse sampling, limited views, restricted bandwidth, heterogeneous media, finite aperture sizes, and insufficient laser power [72].

Antholzer et al. [73] introduced convolutional neural networks (CNNs) for PAI reconstruction in 2017, enhancing reconstruction speed and image quality by learning complex mappings and optimal features. In PACT, DL models yield high-fidelity 3D images [74], and in PAM, they enable high-contrast, high-resolution imaging of biological tissues [75]; and in PAE, they improve clarity and detail by learning rich features from limited datasets [14].

More recently, the landscape of DL in PAI has been reshaped by generative foundation models and self-supervised paradigms, emerging from 2023 to 2025. Recent advances in deep generative models have opened new frontiers in PAI reconstruction. For instance, Song et al. [76] leveraged a diffusion-based prior within a model-based iterative framework to achieve high-quality photoacoustic tomography (PAT) reconstruction from as few as 32 projections, reporting structural similarity index measure (SSIM) and peak signal-to-noise ratio (PSNR) improvements of 0.65 and 5.1 dB over DAS, respectively. Lian et al. [77] introduced a generative prior constrained accelerated iterative reconstruction method that significantly accelerated PAM by reconstructing high-quality images from undersampled data, thereby achieving a single reconstruction time of approximately 5 s. Furthermore, Song et al. [78] proposed a framework utilizing multiple diffusion models for extremely limited-view reconstruction, which integrates multiscale diffusion models to mitigate brightness distortion and structural blurring, significantly enhancing image quality even under severe angular limitations (e.g., 60$^{\circ }$ view).

Moreover, the rise of vision foundation models inspired the development of training-free adaptation strategies for PAI. Deng et al. [79] recently demonstrated that off-the-shelf foundation models (e.g., segment anything model can be directly applied to photoacoustic image processing tasks such as segmentation and enhancement without any task-specific fine-tuning, providing a practical solution to the scarcity of labeled clinical data.

Simultaneously, self-supervised learning frameworks are also gaining momentum. Lan et al. [80] introduced a masked cross-domain self-supervised framework for PACT in which the network learns robust representations by reconstructing masked regions across simulated and experimental domains, thereby eliminating the need for ground-truth images.

Collectively, these emerging approaches leverage the generative power of diffusion models, the generalizability of foundation models, and data efficiency of self-supervised learning to produce physically plausible solutions. This significantly enhances model generalizability while reducing the critical dependence on large, fully annotated datasets.

Despite these promising advances, each paradigm has distinct trade-offs. Diffusion models can achieve high image fidelity even with limited training data but suffer from slow sampling speeds and high computational demands, limiting their use in real-time applications. In contrast, the training-free adaptation of foundation models offers remarkable flexibility and zero-shot capability, yet may struggle with domain-specific features unique to photoacoustic signals, such as wavelength-dependent absorption and acoustic dispersion, potentially compromising quantitative accuracy. While alleviating the need for paired data, self-supervised approaches often rely on carefully designed pretext tasks or simulation-to-real transfer assumptions, which may not be generalizable across heterogeneous tissue types or imaging systems. Crucially, none of these methods currently provide uncertainty quantification or guarantee physical consistency, which are key requirements for clinical deployment. Thus, the choice of reconstruction strategy must be guided not only by image quality metrics but also by practical constraints such as speed, data availability, interpretability, and clinical validation requirements.

### PACT image reconstruction

**Fig. 6:**
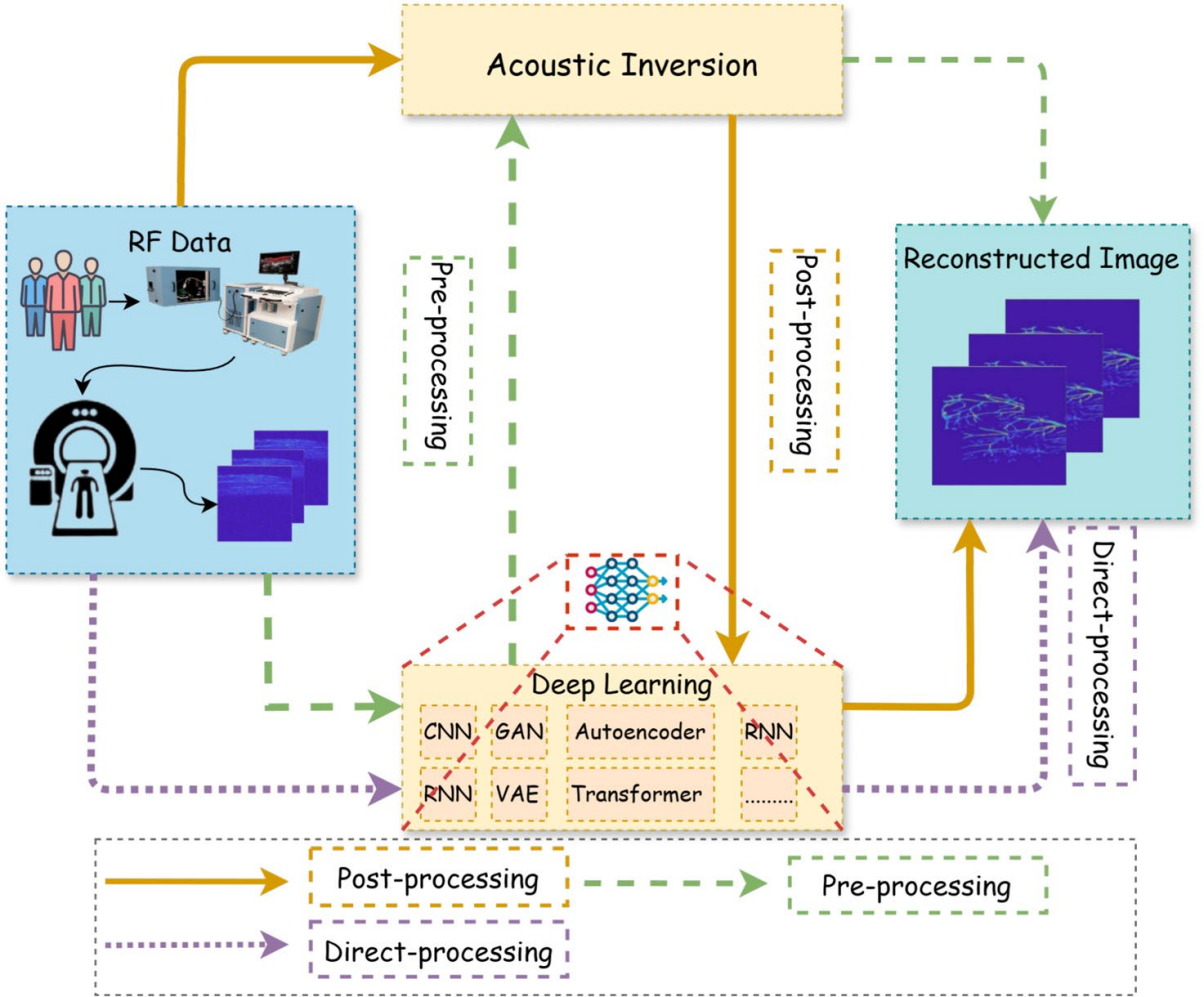
Paradigm illustration using DL for PACT reconstruction

PACT is a well-established preclinical technique that is gaining traction in clinical translation, reconstructing initial pressure distributions from acoustic signals to enable high-contrast, deep-tissue visualization [42]. A key challenge is achieving high SNR images using cost-effective equipment. Sparse sampling often results in a poor reconstruction using artifacts [50, 81]. DL methods have been developed and applied in PACT to enhance the image reconstruction quality, as shown in Table 6 [63, 64, 89–93]. Figure 6 illustrates the integration of acoustic inversion and DL in processing radio frequency data for image reconstruction, highlighting the preprocessing, postprocessing, and direct processing approaches [13].

**Table 6:** Brief summary of DL-based reconstruction approaches for PACT

DL method	Model	Backbone	Key achievement
DL-based reconstruction during pre-processing stage	FC-DNN [82]	A simple fully connected deep neural network that enhances bandwidth by mapping band-limited PA signals to full-bandwidth estimates	On PAT phantom: achieves PC = 0.75/CNR = 2.54 *vs* 0.22/0.01 (limited BW) and 0.32/0.29 (least square deconvolution)
DL-based reconstruction within direct-processing stage	ResUnet [83]	End-to-end ResUnet with residual blocks for direct inversion of PA signals to initial pressure distribution	On numerical and physical phantoms: achieves $>95\%$ higher Pearson correlation and $+39\%$ PSNR *vs* MRR, and $+18\%$ PSNR over UNet++ in simulations
DL-based reconstruction throughout post-processing stage	FD-UNet [84]	Fully dense UNet with enhanced feature transmission and reduced redundant learning for sparse PAT image artifact removal	On mouse brain vasculature dataset: achieves 21.12 ± 1.18 PSNR and 0.65 ± 0.04 SSIM (15 detectors), outperforming UNet by +0.91 PSNR and +0.05 SSIM; gains up to +3.37 PSNR and +0.13 SSIM at 45 detectors
	PAT-AND [85]	Unsupervised artifact disentanglement network with content- and artifact-specific encoders/decoders for unpaired image-to-image translation in sparse-view PAT	On in vivo data under 16 projections: improves SSIM by $\sim$188% and PSNR by $\sim$85% over traditional reconstruction methods
	MDAEP [86]	Model-based iterative reconstruction accelerated by multi-channel denoising autoencoder priors for sparse-view PAT	On in vivo data with 32 projections: achieves $+48\%$ PSNR and $+12\%$ SSIM over U-Net, with significantly accelerated convergence
	Dual-domain Unet [87]	Dual-domain U-Net with Information Sharing Block and mutual information prior for limited-view PAT reconstruction using time- and k-space inputs	On a public clinical dataset: achieves SSIM = $93.56\%$ and PSNR = 20.89 dB, effectively suppressing limited-view artifacts
	NA mechanism [88]	Transformer-based sparse PAT reconstruction with neighborhood attention mechanism and sliding window spatial attention for enhanced local context modeling	Achieves PSNR = 29.39 dB and SSIM = 0.853 at 16 projections, and average PSNR = 31.10 dB/SSIM = 0.878 across 16–64 projections, consistently outperforming UNet, NAFNet, Restormer, and Shuffleformer in extreme sparse PAT reconstruction

#### DL-based reconstruction during pre-processing stage

Data preprocessing employs neural networks to optimize raw data for reconstruction, addressing limitations such as insufficient bandwidth and limited angle data. Specific networks process photoacoustic sinograms, thereby enhancing signal bandwidth and resolution. Gutta et al. [82] utilized a fully connected deep neural network (FC-DNN) to correct sonograms, broaden the effective bandwidth, and increase the SNR of beamformed images by approximately 6 dB. Similar approaches were reported by other researchers [94] and [95].

#### DL-based reconstruction within direct-processing stage

Direct processing in neural networks for PAI involves the use of architectures that map incoming photoacoustic signals directly to the resulting images, bypassing traditional step-by-step processing. Feng et al. [83] introduced an end-to-end Res-Unet model that combined ResNet and Unet architectures to generate high-quality PA images from raw sensor inputs. The model features skip connections and a residual learning mechanism that enhances training efficiency and prevents network degradation. This approach produces images with clear edges and fewer artifacts, simplifying the imaging pipeline and potentially reducing computational requirements. Comparisons with traditional methods and other DL models can provide further insights into their performance and practical implications. This advancement holds promise for enhancing PAI in biomedical applications.

#### DL-based reconstruction throughout post-processing stage

Postprocessing techniques enhance images from conventional algorithms, particularly by addressing the challenges of sparse sampling and limited-angle acquisition in PAI. Antholzer et al. [73] used a U-Net network to refine FBP-reconstructed images under limited-angle conditions. Guan et al. [84] developed a Fully Dense U-Net with dense connections to improve feature transmission. Zhang et al. [87] introduced a dual-domain Unet with an Information Sharing Block to reduce artifacts. Lan et al.’s JEFF-Net [96] employs a DL fusion framework to suppress artifacts, whereas unsupervised methods, such as PA-GAN [97] and PAT-AND [85] address data labeling issues, with PAT-AND performing well under sparse angular conditions.

Physics-informed DL approaches integrate physical models with neural networks. Song et al. [86] proposed a fast sparse reconstruction strategy that combines a multichannel denoising autoencoder prior to a model-based iteration. Guo et al. [98] introduced a high-quality PACT strategy under limited-angle conditions using a fractional diffusion model to enhance imaging quality and speed.

Sparse sampling is crucial for rapid DAQ in PAI; however, reconstructing high-quality images from sparse data is challenging. Chan et al. [88] proposed a neighborhood attention mechanism for sparse PACT reconstruction that emphasized local neighborhood information. Tong et al. [99] developed a feature-projection network (FPNet) for direct reconstruction from sparse and limited-angle data; however, this requires significant computational resources.

#### DL-based reconstruction in hybrid-processing stage

Lan et al.’s Y-Net was a hybrid DL framework that combines direct reconstruction with postprocessing to extract information from raw data and preliminary reconstructed images [100]. Inspired by the U-Net architecture, Y-Net reconstructs the initial PA pressure distribution by optimizing both the raw data and beamformed images using two encoder paths and one decoder path.

### PAM image reconstruction

In PACT and PAM, DL is applied in different ways. In PAM, without requiring inverse reconstruction, DL models directly map input signals to output images, thereby enhancing image quality [3, 73]. This approach effectively addresses the challenges in PAM, such as improving reconstruction, removing artifacts, denoising data, enhancing spatial resolution, and upsampling sparse data. In addition, DL efficiently approximates nonlinear spatial mapping using GPU acceleration. Existing reconstruction algorithms have been continuously refined. This section examines the impact of current DL models on photoacoustic image performance in PAM, focusing on resolution, noise reduction, and imaging depth.

#### DL for resolution enhancement in PAM reconstruction

Spatial resolution is crucial for evaluating photoacoustic images and is influenced by both hardware and reconstruction algorithms. Reducing spatial sampling density and increasing scanning step size in PAM to accelerate imaging degrades resolution and causes aliasing. DL has been applied to lower laser pulse energy and enhance undersampling in PAM.

Recently, a non-pretrained deep iterative prior model was developed to enhance undersampled PAM images and speed up imaging [101]. This model iteratively optimizes fully sampled images from undersampled data using a known downsampling mask, thereby enhancing generalization without the need for paired training. It outperformed interpolation methods and pretrained DL models on mouse brain vasculature and bioprinted gel images without prior training data, marking its first application in PAM.

Seong et al. [102] developed a DL-based method for 3D volumetric reconstruction in PAM, which reduced imaging time and data volume by fully reconstructing undersampled datasets. They adapted SRResNet and validated its performance, achieving the first DL-based 3D reconstruction of undersampled PAM data.

Li et al. [103] introduced PSAD-UNet, a model that enhances transcranial PAI by mitigating bone plate effects, with broad applications in both preclinical and clinical settings. Wang et al. [104] developed PADA U-Net, which reconstructs full images from undersampled data, overcoming the tradeoff between imaging speed and spatial resolution. Shahid et al. [105] proposed BRn-ResNet, which improved the training stability of U-Net and reduced artifacts from sparse data through residual blocks, thereby achieving an SSIM of 0.97.

Cao et al. [106] proposed a mean-reverting diffusion model for AR-PAM by employing an iterative reverse-time SDE to balance imaging depth and lateral resolution, thereby enhancing imaging quality and broadening applicability without sacrificing depth. Le et al. [107] implemented a computational strategy with two GANs for semi-/unsupervised high-resolution sensitive reconstruction in AR-PAM, maintaining imaging capability at enhanced depths. Cheng et al. [75] utilized DL for image transformation to improve deep penetration in OR-PAM.

Zhao et al. [108] proposed a multitask residual dense network to enhance image quality in traditional OR-PAM at ultralow laser doses, incorporating multisupervision learning, dual-channel acquisition, and densely connected layers for richer reconstruction details.

**Table 7:** Brief summary of DL approaches for PAM reconstruction

Aim	Model	Backbone	Key achievement
DL for sparse sampling recovery	DIP [101]	Untrained CNN leveraging deep image prior; iteratively optimizes a full image to match undersampled observation via known downsampling mask	Recovers high-quality PAM images from only 1.4% of fully sampled pixels; outperforms interpolation and rivals supervised DL methods without requiring training or ground truth
	SRResNet [102]	Modified SRResNet with experimentally calibrated subpixel upsampling for 3D isotropic reconstruction from anisotropic sparse PAM volumes	Achieves 80$\times$ faster imaging and 800$\times$ data reduction; outperforms interpolation across PSNR, SSIM, MSE, and 3D vascular fidelity metrics (VOP-MAP, VOP-3D) on real undersampled PAM data
DL-driven resolution enhancement in PAM reconstruction	PADA U-Net [104]	Photoacoustic dense attention U-Net with dense connections and attention gates for enhanced feature recovery in undersampled OR-PAM	At 4$\times$ undersampling, improves PSNR by +2.33 dB and SSIM by +0.117 over bilinear interpolation on bovine bone; enables high-resolution OR-PAM at high speed for bone microstructure imaging
	BRn-ResNet [105]	ResNet enhanced with batch renormalization to stabilize training and reduce subsampling artifacts in sparse-view PAT	Delivers high-resolution PAT reconstructions with SSIM$\approx$0.97, even when trained on low-quality data; significantly improves training stability and artifact suppression under sparse sampling
	Mean-reverting diffusion model [106]	Mean-reverting diffusion model that learns the degradation prior from optical- to acoustic-resolution PAM and recovers high-resolution images via reverse sampling	Achieves more than$3.6\times$ lateral resolution enhancement; improves PSNR by 66% and SSIM by 480% on in vivo AR-PAM data, enabling optical-level resolution with deep penetration
	MT-RDN [108]	Multitask residual dense network with multisupervised learning, dual-channel input, and balanced task weighting for joint denoising, super-resolution, and vascular enhancement	Achieves high-quality OR-PAM at 32$\times$ lower laser dosage (within ANSI limit) and 4$\times$ undersampling; enables real-time, cross-system, cross-anatomy imaging with superior denoising and resolution–first method to jointly resolve the laser dose-speed-quality trilemma
DL for image denoising in PAM reconstruction	UPAMNet [109]	UPAMNet: Unified attention-enhanced CNN with pixel- and perception-level mixed loss for joint PAM super-resolution and denoising	Improves PSNR by +0.59 dB (4$\times$ undersampling), +1.37 dB (16$\times$ undersampling), and +3.9 dB (denoising); generalizes well across multiple PAM datasets with a unified reconstruction framework
	PnP prior [110]	Plug-and-Play framework combining a deep CNN prior with model-based optimization using depth- and frequency-dependent PSF kernels for adaptive AR-PAM enhancement	Outperforms all methods in simulations (best PSNR/SSIM); boosts in vivo SNR from 6.34 to 35.37 and CNR from 5.79 to 29.66; enables a single model to handle diverse AR-PAM degradation conditions via physical PSF integration
DL for imaging depth enhancement in PAM reconstruction	ResUnet-AG [111]	Two-stage residual U-Net with attention gates for adaptive out-of-focus AR-PAM image restoration	Extends AR-PAM depth-of-focus from 1 mm to 3 mm; recovers near-diffraction-limited resolution at 2 mm off-focus, enabling high-quality deep-tissue imaging with standard AR-PAM systems

*DL* Deep learning, *PACT* Photoacoustic compute tomography, *PAT* Photoacoustic tomography, *PA* Photoacoustic, *SSIM* Structural similarity index measure, *PSNR* Peak signal-to-noise ratio; *PAM* Photoacoustic microscopy, *OR*-*PAM* Optical-resolution photoacoustic microscopy, *AR*-*PAM* Acoustic-resolution photoacoustic microscopy, *SNR* Signal-to-noise ratio

**Table 8:** Brief summary of DL-based reconstruction approaches for PAE

Model	Backbone	Key achievement
Focus U-Net [112]	This study incorporated several architectural modifications into the focus U-Net, including the addition of short-range skip connections and deep supervision	Achieves SOTA DSC of 0.941 (CVC-ClinicDB), 0.910 (Kvasir-SEG), and 0.878 (5-dataset combined); 14%–15% improvement over previous SOTA, enabling reliable computer-aided polyp detection
Deep EMI denoising [113]	This study proposed a modified U-Net for EMI denoising in OR-PAE, trained on in vivo rat and rabbit data acquired with 40 MHz transducers	Enables clear 3D reconstruction of ~50 µm capillary networks by removing EMI noise; outperforms classical filters and other CNNs; offers a scalable artificial intelligence solution for low-SNR, low-cost PAE/PAT systems
Contrast limited adaptive histogram equalization [114]	This study proposed a method to enhance endoscopic images by generating three complementary sub-images using contrast limited adaptive histogram equalization, image brightening, and detail enhancement	Significantly improves global and local contrast while suppressing noise amplification, enabling clearer visualization of mucosal and vascular structures in PAE
Approximate Gaussian acoustic field [115]	This study proposed a new photoacoustic/US endoscopic imaging reconstruction algorithm based on the approximate Gaussian acoustic field, which significantly improves the resolution and SNR of the out-of-focus region	Achieves up to 92.6% (simulation) and 52.3% (phantom) improvement in lateral resolution, SNR boost from 16 dB to 36 dB, 8 s/frame reconstruction speed, and validated superior image quality in in vivo rabbit rectal endoscopy
IBP algorithm [116]	This study developed an IBP algorithm for focused detection over centimeter-scale imaging depth, alongside a DL-based algorithm to remove electrical noise	Achieves 0.77/0.65 mm lateral resolution and 25/38 dB SNR at 1.4 cm depth; enables molecular discrimination of ICG in the rabbit rectum; DL denoising eliminates the need for averaging; identifies 800 nm as the optimal wavelength for deep-tissue imaging

*IBP* Improved back-projection, *DL* Deep learning, *PAE* Photoacoustic endoscopy, *OR*-*PAE* Optical-resolution photoacoustic endoscopy, *EMI* Electromagnetic interference, *SNR* Signal-to-noise ratio, *PAT* Photoacoustic tomography, *CNN* Convolutional neural network, *US* Ultrasound

#### DL for image denoising in PAM reconstruction

Noise significantly affects photoacoustic image reconstruction, causing artifacts due to hardware limitations and phase mismatches resulting from inhomogeneous sound velocity and tissue scattering. Zhou et al. [117] introduced a denoising method that combines empirical mode decomposition (EMD) and conditional mutual information, where EMD decomposes noisy signals into intrinsic mode functions, and conditional mutual information aids in denoising.

**Fig. 7:**
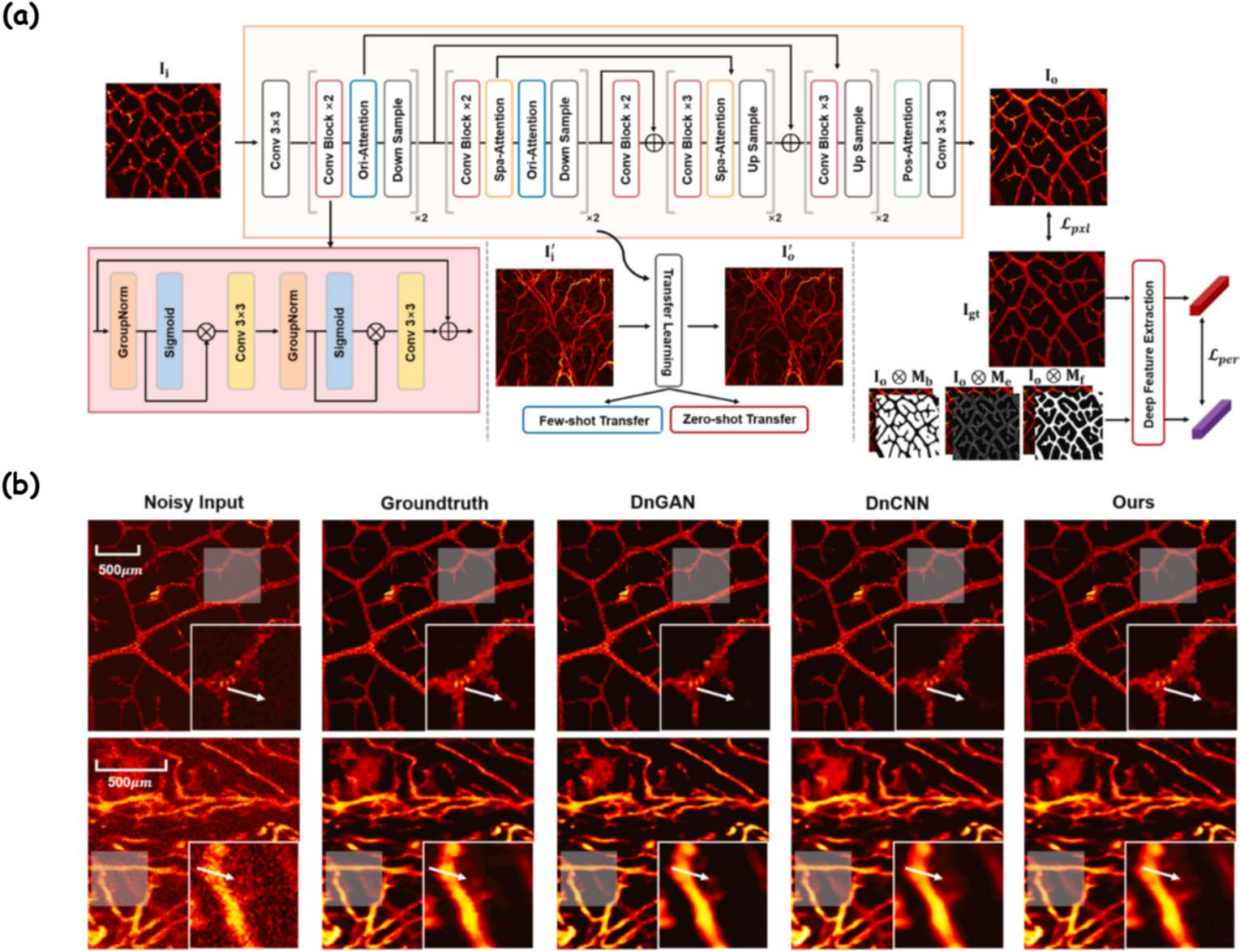
**a** Illustration of UPAMNet architecture, adapted from ref. [109]. The network comprises three modules: feature contraction, feature connection, and feature expansion. Three attention blocks, designed based on deep image priors, are integrated to enhance feature learning. A combined training constraint leveraging semantic segmentation is applied at both pixel and perceptual levels. The inset shows the detailed structure of the ResConv block. Few-shot and zero-shot transfer learning are employed to evaluate generalization on unseen datasets; **b** Denoising results via supervised learning adapted from ref. [109]: first row–Dataset D-I; second row–Dataset D-II. Zoomed views of the white shaded regions (lower right) are shown

Liu et al. [109] proposed UPAMNet, a DL framework that leverages deep image priors to simultaneously address super-resolution and denoising in PAM. By integrating attention-enhanced feature modules and a hybrid training objective combining pixel-wise and perceptual losses, the method achieved notable improvements of 0.59 dB and 1.37 dB PSNR gains for 1/4 and 1/16 undersampled data, respectively, along with a 3.9 dB SNR boost during denoising. More importantly, the model demonstrated strong generalization via few-shot and zero-shot transfers to unseen datasets, indicating its potential for practical clinical deployment. The overall architecture and representative results are shown in Fig. 7.

In PAI, the spatial resolution is degraded by factors such as US attenuation, phase deviations from sound speed variations, and signal waveform broadening. He et al. [118] addressed this issue by proposing an attention-enhanced GAN with an improved U-Net generator to remove noise from PAM images.

However, the low spatial resolution of AR-PAM imaging has limited its adoption [119]. Previous models lacked flexibility or required complex priors and could not adapt to different degradation models. Zhang et al. [110] developed a deep CNN-based model that adaptively handles various degradation functions in AR-PAM image enhancement. This model improves PSNR, SSIM, SNR, and CNR in both simulated and in vivo images by learning vascular image statistics as a plug-and-play prior, demonstrating its interpretability, flexibility, and broad applicability.

#### DL for imaging depth enhancement in PAM reconstruction

In clinical settings, imaging of deep tissues is essential, and neural networks play a key role in enhancing imaging depth.

Traditional AR-PAM systems face challenges such as poor reconstruction in deep or out-of-focus regions. Meng et al. [111] developed ResUnet-AG a residual U-Net with an attention mechanism that enhances deeper tissue imaging by ignoring background noise and extending the depth of field from 1mm to 3 mm. Table 7 summarizes the PAM reconstruction approaches.

###  PAE image reconstruction

Endoscopy is essential for diagnosing internal organ diseases; however, it is often difficult to differentiate microvessels or identify lesions because of internal environmental and imaging conditions [14]. PAE faces challenges owing to limited fields of view and complex in vivo environments. Factors such as blood, illumination variations, specular reflections, and smoke introduce noise, degrading the image quality, particularly in occluded regions. Enhancing the endoscopic image quality by improving details, contrast, brightness, and removing specular reflections is crucial [120].

Yeung et al. [112] introduced focus U-Net, which is a dual attention-gated deep neural network that selectively learns polyp features by incorporating skip connections, deep supervision, and Hybrid Focal Loss to address class imbalance. This network holds promise for noninvasive colorectal cancer screening and other biomedical image segmentation tasks.

In clinical PAE, electromagnetic interference (EMI) noise severely degrades the image quality, particularly in low-cost or miniaturized systems with limited SNR. Gulenko et al. [113] proposed a DL-based algorithm to address this challenge by leveraging a modified U-Net architecture trained on in vivo photoacoustic data from the rat colorectum and rabbit urethra acquired with 40 MHz transducers. Their approach effectively removed structured EMI artifacts while preserving fine vascular structures, outperforming classical filtering methods and alternative CNNs such as SegNet and FCN variants. This method enables high-fidelity 3D visualization of microvasculature, including mesh-like capillary networks ($\sim {50} \upmu$m) and offers a scalable software solution for EMI suppression in emerging light-emitting diode (LED)- or laser diode (LD)-based PAT systems, where hardware-based noise mitigation is impractical.

Building on this, Moghtaderi et al. [114] developed a technique that generates three complementary subimages using contrast-limited adaptive histogram equalization, image brightening, and detail enhancement. These subimages are decomposed via multilevel wavelet transforms and guided filters and then fused with weights to produce a final enhanced image suitable for low-light conditions in endoscopy.

Improving the resolution and SNR in the defocused regions is crucial for designing advanced US/PAE systems. Wang et al. [115] introduced a novel reconstruction algorithm based on an approximate Gaussian acoustic field that enhanced the resolution and SNR in out-of-focus areas. This algorithm was validated using a chicken breast phantom, and in a rabbit rectal endoscopy experiment, it demonstrated improved imaging quality, faster dynamic focusing, and enhanced overall imaging performance.

Molecular PAE imaging of deep tissues presents significant technical challenges. Xiao et al. [116] developed an IBP algorithm for focused detection over centimeter-scale imaging depths and employed a DL-based method to reduce electrical noise from the step motor. These advances have reduced the scanning time and fostered the development of high-penetration molecular PAE. Table 8 summarizes PAE reconstruction approaches.

### Reliability and generalization challenges of DL in PAI

Despite their remarkable performance in controlled settings, DL-based methods in PAI face several critical limitations that hinder robust clinical deployment. A primary concern is overfitting; models trained on limited or non-representative datasets often fail to generalize across different imaging systems, patient populations, or acquisition protocols, leading to degraded performance in real-world scenarios [121]. Moreover, DL approaches are inherently data hungry, requiring large-scale, high-quality, and expertly annotated datasets–resources that remain scarce in PAI, particularly for rare pathologies or multimodal fusion tasks [122]. The “black-box” nature of most DL architectures further complicates their clinical adoption because a lack of interpretability undermines trust and hampers regulatory approval in safety-critical applications [123]. Most critically, when trained on biased or incomplete data, DL models may generate physically implausible reconstructions–such as spurious structures in low-signal regions–that mimic the true pathology but lack biological plausibility [124]. To ensure reliable use, users must validate models on truly independent test sets, monitor performance across diverse data distributions, and employ uncertainty quantification techniques (e.g., Monte Carlo (MC) dropout) to identify low-confidence predictions [125]. Crucially, DL outputs should be cross-validated against physics-based reconstructions or expert interpretations, particularly when clinical decisions depend on the results. Thus, while DL offers transformative potential for accelerating and enhancing photoacoustic image reconstruction, its clinical integration demands rigorous validation, transparency, and a clear understanding of its inherent limitations. The clinical adoption of DL-enhanced PAI also depends on practical factors such as laser safety, acoustic coupling stability, imaging speed, and system cost. Although these operational barriers are well-documented in the literature [19, 20], they underscore that even a perfectly robust DL model cannot succeed in isolation from a broader imaging ecosystem.

## DL-based quantitative analysis in PAI

Although PAI excels in generating high-contrast images, its translation from a qualitative technique to a reliable clinical diagnostic tool hinges on accurate quantitative analysis. The accurate quantification of physiological parameters–such as blood oxygen saturation (sO$_2$)–is critical for clinical decision-making in oncology, vascular diseases, and neurology.

PAI provides comprehensive structural, functional, molecular, and kinetic information by leveraging endogenous chromophores–such as hemoglobin, lipids, melanin, and water–as well as exogenous contrast agents, including clinically approved dyes such as ICG and methylene blue. A key feature of PAI is its ability to differentiate deoxyhemoglobin (Hb) from oxyhemoglobin (HbO$_2$), enabling noninvasive mapping of blood oxygen saturation (sO$_2$)–crucial for detecting conditions such as ischemia, hypoxia, and hypoxemia [126]. Furthermore, PAI utilizes the strong optical absorption of melanin to visualize melanomas and other pigmented lesions, offering valuable diagnostic insights [49].

**Fig. 8:**
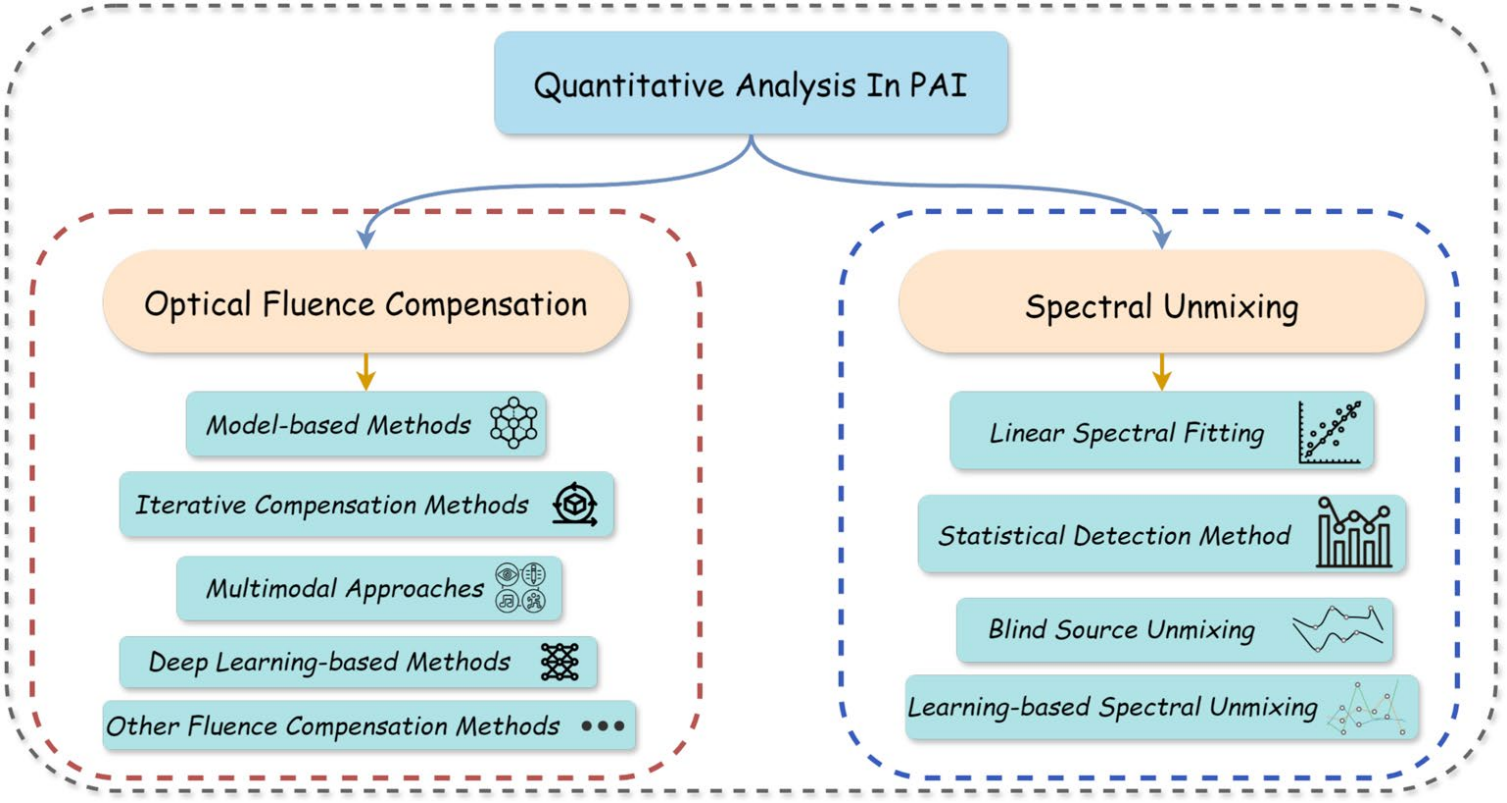
Overview of quantitative analysis of PAI. PAI: Photoacoustic imaging

However, accurate interpretation of this rich contrast is hindered by the spatially heterogeneous optical fluence in biological tissues and significant spectral overlap among chromophores. Consequently, robust quantitative analysis is essential and typically requires optical fluence compensation and spectral unmixing to recover accurate chromophore concentrations [127]. This subsection focuses on the pivotal role of DL in addressing these fundamental challenges, as summarized in Fig. 8, enabling robust and clinically relevant quantitative photoacoustic imaging (qPAI).

### Optical fluence compensation

The accuracy and clinical utility of qPAI are fundamentally limited by the spatial distribution of optical fluence, which varies with tissue depth and illumination wavelength. Optical fluence compensation mitigates quantitative inaccuracies arising from inhomogeneous light distribution, thereby enhancing the accuracy of estimating physiological parameters, such as hemoglobin concentration and blood oxygen saturation (sO$_2$). DL models can be trained to learn complex feature representations from raw photoacoustic data and directly estimate the underlying optical fluence, thereby enabling data-driven compensation for variations in tissue optical properties. This data-driven approach enables automatic and efficient fluence compensation, thereby significantly enhancing the quantitative accuracy of reconstructed PA images. Although various fluence compensation strategies exist–including model-based, iterative, and multimodal approaches–DL-based methods have recently emerged as powerful data-driven alternatives [127].

#### Model-based methods

Several light transport models have been employed to estimate the optical fluence, including the diffusion equation (DE) and MC simulations, which numerically solve the radiative transfer equation [128] and diffusion dipole model [129]. The DE is well-suited for macroscopic fluence estimation in highly scattering tissues, as demonstrated by Zhao et al. [130], who utilized finite element analysis (FEA) to generate fluence maps. MC simulations employed by Kirillin et al. [131] and Hirasawa et al. [132] stochastically modeled photon propagation and effectively mitigated spectral coloring artifacts caused by wavelength-dependent light attenuation. Ranasinghesagara et al. [133] employed multi-illumination photoacoustic microscopy to estimate the tissue optical properties, which were subsequently used as inputs to an MC model for fluence calculation.

#### Iterative compensation methods

Iterative fluence compensation estimates the optical fluence distribution by solving an inverse problem; starting from an initial guess, it iteratively updates the fluence to minimize the discrepancy between the measured and simulated photoacoustic signals until convergence. Unlike model-based approaches, iterative methods do not require prior knowledge of the tissue optical properties, thereby avoiding potential biases introduced by inaccurate parameter assumptions. Consequently, they often exhibit greater robustness in heterogeneous or previously uncharacterized tissue environments. These algorithms are well suited for complex, heterogeneous media but are computationally intensive–particularly for 3D datasets or when coupled withMC light transport models [134]. While reducing the computational cost is desirable, achieving higher quantitative accuracy often requires additional iterations or more sophisticated optimization schemes, highlighting the inherent trade-off between accuracy and computational efficiency.

#### Multimodal approaches

Multimodal integration is pivotal for the clinical translation of PAI, as it synergistically combines complementary modalities to overcome the limitations of any single technique, which cannot simultaneously provide high-resolution anatomical detail, deep tissue penetration, functional specificity, and molecular sensitivity. The most established configuration integrates PAI with US, enabling co-registered anatomical (US) and functional/molecular (PAI) imaging using a shared transducer array. This design underpins the commercial systems for breast and thyroid imaging. Recent advancements in lightweight handheld scanners have enabled clinical panoramic volumetric photoacoustic/US imaging, facilitating 3D visualization of vascular anatomy, hemodynamics, and oxygen saturation over large fields of view [135].

Beyond US, hybrid platforms incorporating OCT deliver micrometer-scale structural information in superficial tissues (e.g., skin, retina), complementing PAI’s hemodynamic contrast for dermatological and ophthalmic applications. For deep-tissue imaging, integration with magnetic resonance imaging (MRI) leverages superior soft-tissue contrast to guide PAI reconstruction and interpretation, particularly in neuro-oncology. Emerging systems incorporating fluorescence lifetime imaging enable multiparametric mapping of absorption- and fluorescence-based biomarkers in a single acquisition.

**Fig. 9:**
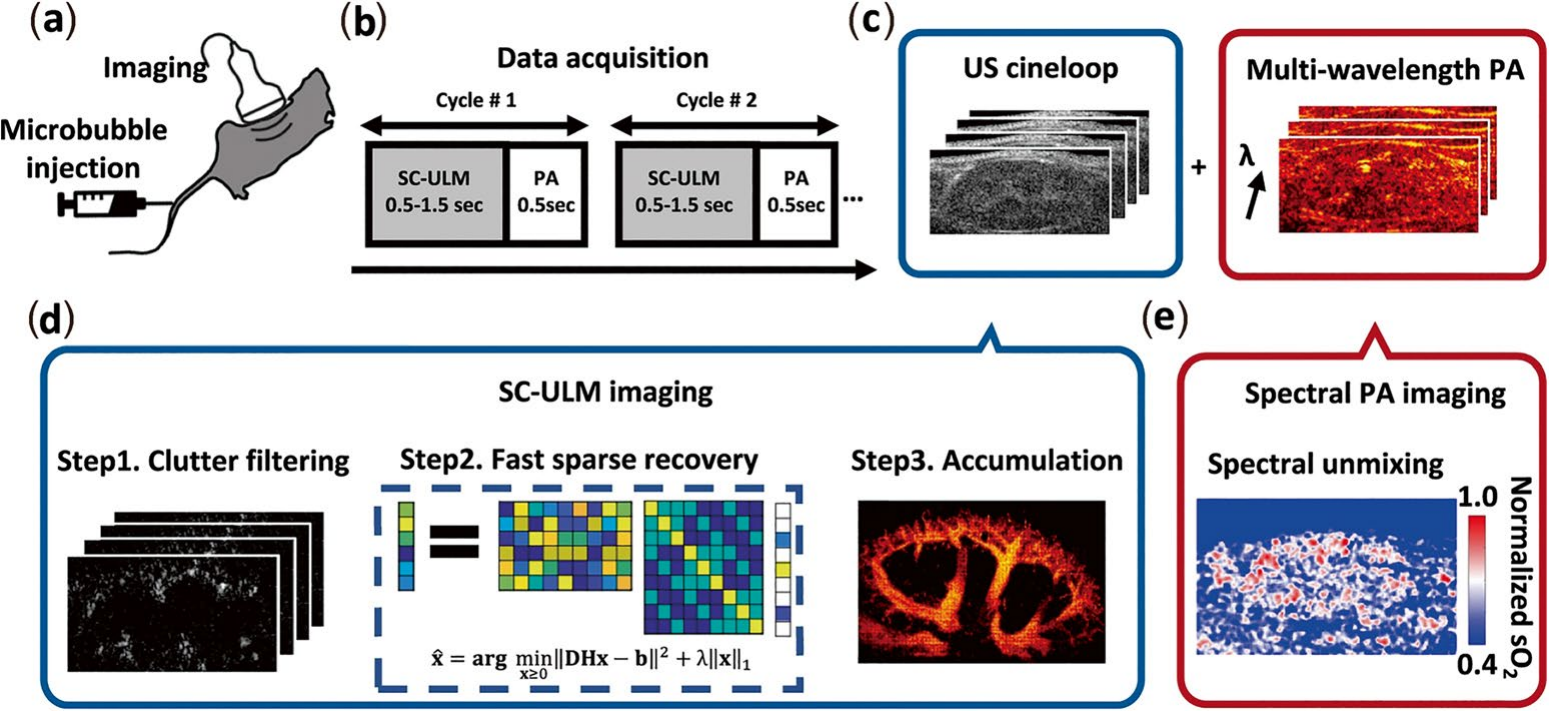
Representative multimodal imaging workflow combining photoacoustic and super-resolution ultrasound (US), entirely adapted from Zhao et al. [136]. **a** Animals are injected with a microbubble solution before (or during) imaging. **b** During data acquisition (DAQ), multi-wavelength photoacoustic (PA) and plane-wave fast US imaging are recorded alternately at each position; the minimum DAQ duration for US localization microscopy (ULM) is determined by the pixel saturation curve characterization time. **c** US cine-loops and multi-wavelength PA images are stored and processed separately via the pipelines shown in (**d**) and (**e**). **d** Sparsity-constrained (SC)-ULM imaging involves three steps: clutter filtering, sparse recovery over frames, and location accumulation. **e** Blood oxygen saturation (sO$_2$) maps are generated through linear spectral unmixing of multi-wavelength PA images

To concretely illustrate how such hybrid photoacoustic/US systems operate in practice, Fig. 9 shows a representative multimodal imaging workflow adapted from Zhao et al. [136]. This example demonstrates the synchronized acquisition and parallel processing of photoacoustic and super-resolution US data, enabling the simultaneous mapping of microvascular perfusion and blood oxygen saturation within a unified imaging framework.

From a quantitative perspective, auxiliary modalities enhance the accuracy of PAI-derived biomarkers. US-derived structural priors constrain fluence models to improve the quantification of oxygen saturation (sO$_2$), whereas diffuse optical tomography (DOT) provides 3D optical property maps for deep-tissue fluence correction. However, these approaches require additional hardware, labor-intensive segmentation (e.g., manual or artificial intelligence-assisted delineation), and substantial computational resources–particularly for 3D implementations. Despite these challenges, the synergistic value of multimodal systems in delivering comprehensive diagnostic information within clinical workflows underscores their critical role in the regulatory approval and clinical translation of PAI. In addition, calibration techniques such as reference phantom normalization and wavelength-dependent fluence correction are crucial to ensure accurate quantitative assessment.

#### DL-based methods

DL offers a data-driven framework for optical fluence compensation in qPAI. For instance, Yang et al. [126] employed a deep residual recurrent U-Net (DR2U-Net) to estimate sO$_2$ from photoacoustic data. However, the accuracy and generalizability of DL models depend heavily on the availability of large and diverse training datasets. Moreover, training such models requires substantial computational resources and time, whereas complex architectures may incur high inference latency.

To mitigate these challenges–particularly data scarcity and limited generalizability–recent advances have integrated physical priors and leveraged multimodal data. Physics-informed DL frameworks now enable end-to-end quantitative reconstruction of absorption coefficients while considering the underlying photoacoustic physics [137]. Furthermore, models trained on co-registered US and photoacoustic data can estimate spatially varying fluence for improved sO$_2$ quantification [138]. Self-supervised methods have demonstrated robust artifact reduction in clinical settings without requiring paired training data [80].

#### Other fluence compensation methods

In addition to iterative and model-based approaches, alternative strategies, such as wavefront shaping and algorithmic correction, have been developed to address optical fluence variations. For instance, Caravaca-Aguirre et al. [139] demonstrated the use of a spatial light modulator (SLM) to modulate the wavefront of a 532 nm laser, enabling focused light delivery through a scattering medium (a glass diffuser) onto capillary targets. Complementarily, Fadhel et al. [140] proposed a fluence matching algorithm designed to correct errors in raw photoacoustic images caused by wavelength-dependent fluence variations, thereby improving the accuracy of sO$_2$ measurements. These methods represent complementary strategies for mitigating fluence-related artifacts and enhancing the quantitative accuracy of PAI.

### Spectral unmixing

Spectral unmixing is a technique that decomposes a mixed photoacoustic signal within each pixel into contributions from individual chromophores–such as oxy- and deoxyhemoglobin, lipids, and melanin–by determining the spectral contribution of each endmember to the observed spectrum [141]. Photoacoustic data are typically acquired across multiple wavelengths, yielding multispectral images that encapsulate the overlapping absorption from these endmembers [142, 143]. Despite the complexity of tissue composition, spectral unmixing has proven effective in distinguishing major chromophores.

Applying DL to spectral unmixing can further enhance the separation of absorbers by learning complex nonlinear relationships in multispectral data, thereby enabling a more accurate quantitative analysis. Nevertheless, conventional spectral unmixing methods remain challenged by wavelength-dependent fluence variations, spectral cross-talk, and the lack of reliable calibration standards.

#### Linear spectral fitting

Traditional approaches to spectral unmixing in PAI typically rely on linear regression using the linear mixing model, which assumes that the measured signal at each pixel is a linear combination of the known absorption spectra of the constituent chromophores weighted by their respective concentrations [144]. For example, Feng et al. [145] applied linear spectral unmixing to multi-wavelength photoacoustic data to isolate collagen as a biomarker for bone health assessments. Despite its widespread use, conventional linear spectral fitting is limited by the need for accurate prior knowledge of chromophore absorption spectra and wavelength-dependent fluence variations, which distort spectral signatures in complex or deep tissues [143].

#### Statistical detection methods

Statistical detection methods aim to identify specific target chromophores with known absorption spectra–such as exogenous nanoparticles, reporter genes, and oxy- and deoxy-hemoglobin–without requiring a priori knowledge of the background absorption spectra. For example, Tzoumas et al. [146] introduced eigenspectra multispectral optoacoustic tomography (eMSOT) to estimate sO$_2$ levels in vivo. Nevertheless, these approaches typically demand dense spectral sampling and high signal fidelity, which can increase computational complexity and pose practical challenges in dynamic in vivo imaging scenarios.

#### Blind source unmixing

Blind source unmixing (BSU) offers an alternative paradigm for qPAI that circumvents the need for prior knowledge of chromophore absorption spectra–a key limitation of conventional linear unmixing. Unlike model-based approaches, BSU aims to simultaneously estimate unknown spectral signatures and their spatial concentration maps directly from multispectral photoacoustic data, typically by exploiting statistical or structural properties, such as source independence, sparsity, or non-negativity [147]. This makes it particularly attractive for analyzing complex biological tissues, for which complete spectral priors are unavailable. However, its practical application in PAI remains challenging because of wavelength-dependent fluence variations, strong spectral correlations among endogenous chromophores (e.g., oxy- and deoxyhemoglobin), and the limited number of available excitation wavelengths.

#### Learning-based spectral unmixing

Kirchner et al. [148] developed context-enhanced quantitative photoacoustic imaging using MC simulations and random forests to enhance spectral unmixing. Cai et al. [149] introduced an end-to-end ResUnet approach for accurate quantitative imaging using simulations. Gröhl’s team used learned spectral decoloring with a neural network to improve oxygenation estimation accuracy [149]. Olefir et al. [150] enhanced MSOT with DL-eMSOT, achieving superior performance using synthetic data.

To address the challenges of qPAI, it is essential to account for spatial variations in optical fluence to derive accurate absorption maps. Strategies include model-based methods, iterative compensation, multimodal approaches, and DL techniques. Robust spectral unmixing is critical to obtaining accurate physiological and biochemical information at depth. Recent advancements such as linear spectral fitting, statistical detection methods, BSU, and learning-based approaches have significantly improved the decoding of mixed-pixel information into distinct chromophores. Notably, the challenges in qPAI are often interconnected owing to the hybrid nature of optical absorption and acoustic detection, which is beneficial for developing effective strategies to enhance the reliability and applicability of qPAI across various biomedical contexts.

### Emerging excitation and computational strategies in PAI

Recent progress in PAI stems from synergistic advances in light delivery, computational reconstruction, and wavefront control. These innovations address the fundamental trade-offs in PAI–between depth and resolution, speed and fidelity, cost, and performance–accelerating its translation to preclinical and clinical applications. Emerging strategies fall into two complementary categories: (1) novel excitation sources that enhance spectral coverage and practicality and (2) advanced computational and light-field methods that improve image quality and effective optical focusing.

#### Novel excitation sources

Conventional PAI systems often rely on tunable optical parametric oscillators (OPOs) or Q-switched lasers, which deliver high pulse energy but are bulky, expensive, and limited in wavelength agility. Next-generation light sources overcome these constraints by enhancing tissue penetration, expanding the spectral range, and enabling compact system design.

Lasers operating in the second near-infrared window (NIR-II, 1000–1700 nm) leverage reduced tissue scattering and absorption within this band, enabling deep-tissue imaging with high spatial resolution, as demonstrated in breast tomography and transcranial vascular mapping [151, 152]. Combining NIR-II excitation with exogenous contrast agents, such as semiconducting polymers, further extends molecular imaging capabilities.

Supercontinuum sources, generated by pumping photonic crystal fibers with ultrashort pulses, provide broad emission spectra and enable rapid, all-electronic wavelength tuning, eliminating moving parts. These sources support real-time multispectral imaging to quantify key chromophores, including hemoglobin, lipids, and water [153–155].

High-power LEDs and LDs offer compact, cost-effective alternatives that can be engineered for eye-safe operation. Advances in array architectures, beam shaping, and signal averaging have enabled portable PAI systems that are suitable for point-of-care diagnostics and wearable monitoring [156]. Together, these sources broaden the applicability of PAI from deep tissue organ imaging to decentralized health monitoring.

#### Advanced computational and light-field techniques

Computational methods in PAI integrate physical models with data-driven priors to mitigate challenges such as limited-view detection, sparse sampling, and measurement noise. Model-based iterative reconstruction combined with DL–including unrolled networks and diffusion models–enables high-fidelity image recovery while reducing acquisition time and hardware complexity [157, 158]. Self-supervised and transfer-learning strategies further facilitate quantitative imaging in settings where ground truth annotations are unavailable.

Light-field modulation techniques, employing SLMs, digital micromirror devices, or programmable metasurfaces, allow precise control over the optical wavefront. Structured illumination suppresses the out-of-focus background, enhancing contrast in scattering media [159]. Meanwhile, wavefront shaping and time-reversed ultrasonically encoded focusing can confine the optical excitation to spots smaller than the acoustic diffraction limit, thus enabling high-resolution imaging deep within biological tissues [10].

The integration of advanced light sources, adaptive illumination, and computational reconstruction represents a paradigm shift for PAI–transforming it from a passive imaging modality into an active, closed-loop sensing platform. This evolution requires unified systems that synergistically combine programmable excitation, high-speed computation, and intelligent feedback to drive its clinical adoption and enable precise diagnostics.

## Future work

Although clinical applications of PAI have been extensively explored, and the International Photoacoustic Standardization Consortium has advocated for standardization in data management, hardware testing, and clinical protocols, PAI still faces significant barriers to routine clinical adoption. Despite receiving regulatory approval from the FDA and CE, as well as integration into the DICOM standard [7], a critical evaluation reveals persistent technical and physical constraints.

First, laser safety is strictly governed by maximum permissible exposure limits, such as those defined in ANSI Z136.1, which restrict optical fluence and consequently limit imaging depth, particularly in pigmented or highly absorbing tissues. Second, reliable acoustic coupling between transducers and human skin remains challenging owing to anatomical curvature, patient motion, hair, dependence on US gel, and factors that compromise sterility, hinder wearable deployment, and impede longitudinal monitoring. Third, despite advances in artificial intelligence-accelerated reconstruction, current imaging speeds are often insufficient for real-time guidance in dynamic clinical scenarios, such as cardiac or interventional procedures.

Beyond these technical limitations, broader clinical and operational hurdles further impede translation. The high cost of PAI systems–driven by tunable lasers, dense US arrays, and hybrid imaging architectures–restricts their deployment to well-funded academic centers, limiting accessibility in point-of-care or resource-constrained settings. Moreover, the absence of universally accepted acquisition protocols and formalized operator training programs undermines reproducibility and diagnostic consistency across institutions and users. Although ongoing innovations in hardware, software, and artificial intelligence hold considerable promise, these intertwined practical, regulatory, and systemic challenges must be addressed systematically to enable broad clinical adoption.

Looking beyond current limitations, three transformative frontiers are poised to redefine the clinical impact of PAI. First, artificial intelligence-enhanced PAI enables not only accelerated and artifact-robust reconstruction but also high-fidelity spectral unmixing of multiple contrast agents, which is critical for multiplexed molecular imaging and quantitative biomarker extraction. Second, the emergence of wearable and flexible PAI probes holds promise for continuous, noninvasive monitoring of hemodynamics, tissue oxygenation, and drug kinetics in ambulatory settings, although challenges in motion robustness and long-term acoustic coupling persist. Third, theranostic integration is advancing toward closed-loop interventions, in which PAI guides and monitors photothermal therapy or triggers photoacoustic-controlled drug release from stimuli-responsive nanocarriers in real time. Together, these directions bridge diagnostic imaging with personalized therapy, positioning PAI as an active enabler of precision medicine rather than merely a passive observation tool.

### Next-generation excitation sources and system miniaturization

Recent developments in excitation technologies have driven the evolution of PAI toward more compact, efficient, and spectrally versatile systems. Although conventional OPOs and Q-switched lasers provide high pulse energies, their size, cost, and limited tunability have motivated the exploration of alternative sources, including NIR-II lasers, supercontinuum sources, and high-power LEDs or LDs. These emerging sources facilitate deeper tissue penetration, broadband tunability, and improved energy efficiency, enabling miniaturized, wearable, and point-of-care PAI implementation.

Furthermore, the combination of adaptive illumination and computational reconstruction, in which excitation is modulated in response to tissue feedback, represents a paradigm shift. This approach allows subacoustic-resolution imaging while maximizing the efficiency of compact light sources. Collectively, these advances have made PAI systems faster, smaller, and more accessible, accelerating their transition from experimental research to clinical applications.

### Single image super-resolution in PAI

High-resolution photoacoustic images are essential for clinical applications and image analysis; however, hardware limitations often necessitate trade-offs in scan time, SNR, and spatial coverage. DL-based super-resolution techniques, which have been extensively researched in other modalities, enhance photoacoustic image reconstruction through iterative optimization of the gradient flow, improve model stability, and capture detailed information. These techniques integrate dense connections and feedback mechanisms to leverage prior information for self-correction, leading to stable iterative optimization and accelerated model convergence, thus improving reconstruction fidelity.

Implementing super-resolution techniques in PAI enhances diagnostic value and analytical precision without requiring costly hardware upgrades. Advanced DL models tailored for super-resolution tasks enable higher-fidelity reconstruction, offering clearer visualization of anatomical structures and potential pathologies. Since the pioneering work of Dong et al. [160] in 2014 , optimized models such as SRCNN [161], SRGAN [162], CAMixerSR [163], and CoSeR [164] have emerged, showing significant progress within the super-resolution technology domain that is specifically applicable across medical imaging contexts. Furthermore, deep-structured networks such as VDSR [165], DRRN [166], and RFDN [167] combined with innovative frameworks exemplified by deep multiscale networks [168] have further enhanced the quality metrics associated with existing SR methods utilized throughout healthcare settings.

### Physics-informed reconstruction in PAI

Using DL for PAI presents challenges, such as managing incomplete datasets and high computational costs associated with large training requirements. To address these issues, physics-informed DL approaches have been developed. These methods integrate physical models with artificial intelligence to enhance reconstruction quality and reduce processing time. By incorporating physical constraints and prior knowledge, these approaches can improve the performance and robustness of DL architectures. Notable examples include PAT-MDAE [86] and a physics-driven DL-based FBP framework [169], which demonstrate significant potential for advancing biomedical PAI applications.

### DL-based multimodal fusion in PAI

Multimodal imaging systems, combining PAI with OCT, US, and other modalities, enhance imaging depth and spatial resolution, providing comprehensive tissue information [170]. Multimodal image-guided surgical navigation, integrating techniques such as endoscopy, US, and fluorescence, improves visualization and spatial identification of critical structures, supporting minimally invasive procedures in neurosurgery, orthopedics, and vascular interventions. Key technologies include multimodal image segmentation, surgical planning, position calibration, image registration, and multi-source information fusion. Recent advancements in image fusion techniques, such as GANMcC [171], leverage GANs alongside Transformers to effectively integrate gradient and intensity information. Nyayapathi et al. [172] proposed a novel architecture that integrates both Transformer and CNN elements, featuring global and local branches within the encoder module. Furthermore, the combination of photoacoustic/US systems with methods like DOT and MRI enhances disease monitoring and diagnosis; this is particularly beneficial for brain imaging aimed at obtaining functional and molecular insights through MRI. Anatomical data derived from US improves the lateral resolution of PAI systems, enabling detailed visualization of cerebral vasculature [173]. Additionally, it is crucial to evaluate these fusion technologies in clinical settings to ensure their continued advancement [174].

### Quantitative analysis in PAI

Future research on qPAI will focus on enhancing luminous flux modeling for precise tissue absorption coefficient reconstruction, improving spectral unmixing using advanced methods, and developing unsupervised super-resolution models to address data scarcity. Efforts will also include creating compact and efficient network models, innovating evaluation metrics aligned with human perception, and deepening DL theory for better reliability and interpretability. Advances in reconstruction techniques will address limited views and acoustic heterogeneity while enabling real-time reconstruction using DL. Integrating qPAI with other modalities improves resolution and contrast. Clinical trials will establish its efficacy and safety, and new contrast agents, portable systems, and functional/molecular imaging extensions will further advance qPAI in biomedical research and clinical diagnosis [175].

## Conclusions

While DL has substantially advanced PAI, improving reconstruction speed, spatial resolution, and quantitative accuracy, these advances entail inherent trade-offs. As highlighted in this review, challenges such as overfitting, limited training data, and poor model interpretability continue to hinder clinical translation. Moving forward, algorithmic development must prioritize not only performance but also robustness, transparency, and reproducibility. PAI has emerged as a powerful hybrid imaging modality, leveraging optical contrast and US resolution to overcome shallow penetration and strong scattering that limit purely optical techniques such as OCT. Over the past decade, PAI has shown considerable potential in disease screening, diagnosis, and longitudinal monitoring across diverse organ systems. This review outlines the fundamental principles of PAI and its advantages over conventional optical imaging and US, examines the limitations of traditional reconstruction and analysis methods, and emphasizes the transformative role of DL in advancing PACT, PAM, and PAE. Artificial intelligence-driven approaches have proven particularly effective in mitigating image degradation caused by sparse spatial sampling, which is a common constraint in practical PAI systems. By combining physics-informed architectures with data-driven priors, these methods reduce artifacts, improve structural delineation, enhance source localization, and enable reliable quantification of optical absorption. Nevertheless, two major challenges persist: compensating for wavelength- and depth-dependent optical fluence to obtain accurate absorption maps and achieving robust spectral unmixing for precise physiological and molecular characterization at depth. Addressing these challenges is essential to unlock the clinical potential of PAI. In summary, this review identifies several promising research directions, including single-image super-resolution, physics-informed DL, multimodal image fusion, and end-to-end quantitative biomarker analysis. Together, these pathways illustrate how DL continues to reshape and extend the capabilities of PAI, paving the way for broader clinical impact.

## Data Availability

Not applicable.

## Abbreviations

PAI: Photoacoustic imaging
DL: Deep learning
3D: Three-dimensional
US: Ultrasound imaging
ICG: Indocyanine green
OCT: Optical coherence tomography
IVPAI: Intravascular photoacoustic imaging
PACT: Photoacoustic compute tomography
PAM: Photoacoustic microscopy
PAE: Photoacoustic endoscopy
OR-PAM: Optical-resolution photoacoustic microscopy
AR-PAM: Acoustic-resolution photoacoustic microscopy
TR: Time reversal
DAS: Delay-and-sum
FBP: Filtered back projection
SNR: Signal-to-noise ratio
SSIM: Structural similarity index measure
PSNR: Peak signal-to-noise ratio
EMD: Empirical mode decomposition
EMI: Electromagnetic interference
qPAI: Quantitative photoacoustic imaging
DE: Diffusion equation
MC: Monte Carlo
MRI: Magnetic resonance imaging
DOT: Diffuse optical tomography
DAQ: Data acquisition
ULM: Ultrasound localization microscopy
SC-ULM: Sparsity-constrained-ultrasound localization microscopy
SLM: Spatial light modulator
BSU: Blind source unmixing
OPO: Optical parametric oscillator
LED: Light-emitting diode
LD: Laser diode
US: Ultrasound
eMSOT: Eigenspectra multispectral optoacoustic tomography
